# Multiscale Simulation of Primary Charge Separation Mechanisms in an LH1-RC Complex

**DOI:** 10.1021/jacsau.5c01095

**Published:** 2025-10-23

**Authors:** Sayan Maity, Ulrich Kleinekathöfer

**Affiliations:** † Department of Physics and Astronomy and Thomas Young Centre, 4919 University College London, London WC1E 6BT, U.K.; ‡ School of Science, 84498 Constructor University, Campus Ring 1, 28759 Bremen, Germany

**Keywords:** photosynthesis, purple bacteria, reaction centers, charge separation, MD, QM/MM, TD-DFT/B

## Abstract

The light-harvesting complex II (LH2) of purple bacteria captures solar energy using bacteriochlorophyll (BChl) pigments. This energy is then transferred to the LH1 complex and subsequently to the embedded reaction center (RC). The initial separation of charges instigates a series of subsequent processes, ultimately culminating in the synthesis of adenosine triphosphate (ATP). While the excitation transfer process within the LH2 complex has been thoroughly characterized, the atomistic mechanism of charge separation in the RC remains unresolved. In this study, we employed a combination of classical molecular dynamics (MD), ab initio quantum mechanics/molecular mechanics (QM/MM) MD, and time-dependent density functional theory (TD-DFT) to delineate the excitation funnel within the LH1 ring of *Thermochromatium tepidum*, which is instrumental in facilitating charge separation in the RC. The analysis of the excitation profile indicates that the process of charge separation does not originate from the so-called “special pair” (P), but rather from the adjacent P/B BChl pair located on the active branch. The protein environment has been determined to play a pivotal role in this process. A competing low-lying charge-transfer state on the inactive branch exhibits inconsistent directionality, thereby rendering it an inefficient route. This study presents the first comprehensive analysis of an entire LH1-RC complex, and the findings challenge traditional models, highlighting the role of the protein scaffold as a crucial factor for charge separation in bacterial photosynthesis.

## Graphical abstract

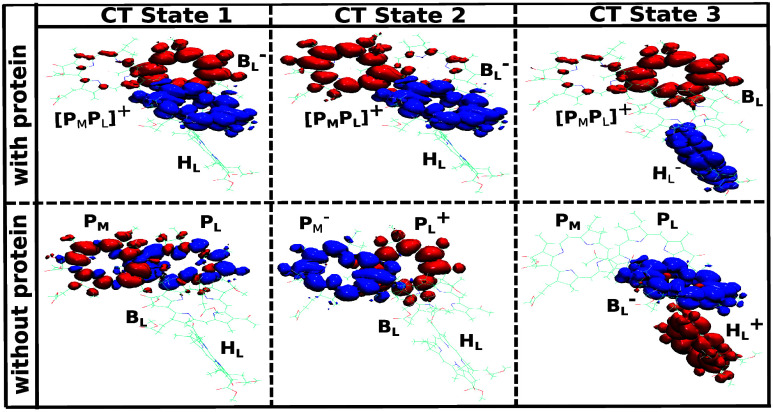

## 1 Introduction

Photosynthesis is one of the fundamental mechanisms by which the sun’s energy is efficiently converted into chemical energy and occurs in various organisms such as plants, bacteria, and algae.^1^ This intricate process involves the utilization of chlorophyll, bacteriochlorophyll (BChl), bilin, or similar pigment molecules that are incorporated within a protein scaffold known as the light-harvesting antenna complexes. These complexes play a crucial role in optimizing the photosynthetic mechanism. The LH complexes capture the sunlight and transfer the energy to neighboring complexes in the form of excitons, i.e., electron–hole pairs. Eventually, this energy is transported to the reaction center (RC) of the respective photosystem. At the RC, the electron–hole pair gets split into separate electrons and holes, which are used in further steps of photosynthesis. In plant photosynthesis, the absorption of sunlight energy primarily occurs within the major light-harvesting complex known as LHCII.^2–4^ Subsequently, this energy is transmitted to the minor complexes and ultimately reaches the RC to initiate the charge operation. Similarly, in the case of purple bacteria, solar energy is initially captured by the peripheral light-harvesting antenna LH2. It is then transferred to the LH1 complex, which directs the energy to the reaction center.^5^ In the RC, this process triggers a separation of charges and a redox event, producing a proton-motive force.^6^ There are two distinct varieties of LH1-RC complexes within purple bacteria: one takes the form of an S-shaped dimeric unit, while the other is an elliptical monomeric unit.^7^ In the S-shaped LH1-RC complex, PufX transmembrane polypeptides are strategically located at the heart of the LH1-RC dimer, facilitating a tightly interlocked association between the components and mediating the dimerization of LH1-RC. Recent characterizations through cryo-electron microscopy (cryo-EM) have revealed several structures,^8–11^ but the particular assembly process of the S-shaped LH1-RC dimer remains elusive. The purple bacteria RC complex (PbRC) is located either within the open S-shaped form or in the closed elliptical form of an LH1 complex, comprising a combination of 14 to 17 α (inner) and β (outer) polypeptidesubunits.^9,10,12–15^ Each α or β polypeptide contains BChl and carotenoid molecules. The PbRC consists of *L*, *M*, and *H* subunits, occasionally accompanied by an additional subunit called the cytochrome (Cyt) subunit. The *L* and *M* branches of PbRC are active and inactive pathways and are also referred to as *A* and *B* branches in literature.^16^ Within the *L* and *M* subunits, there is a special pair of BChl molecules, namely P_*L*_ and P_*M*_, together with two additional BChl molecules, name B_*L*_ and B_*M*_, two bacterio-pheophytins (BPh), i.e., H_*L*_ and H_*M*_, a carotenoid and electron acceptors referred to as primary and secondary quinones, denoted Q_*A*_ and Q_*B*_, respectively. Overall, the RC has *C*_2_ symmetry for the pigment network as depicted in Figure 1. At the end of this electron transfer chain, Q_*B*_ takes up two electrons and two protons from the cytoplasmic side of the membrane, undergoing a reduction to form Q_*B*_H_2_. Subsequently, the Q_*B*_H_2_ traverses the LH1 complex, moving toward the quinone pool located within the photosynthetic membrane. This movement facilitates the transfer of the reducing power to the Cytochrome *b*/c1 complex while releasing protons on the periplasmic side. Although direct experimental evidence is limited, it is believed that this quinone transport mechanism occurs in most, if not all, purple bacteria.^6^

**Figure 1:**
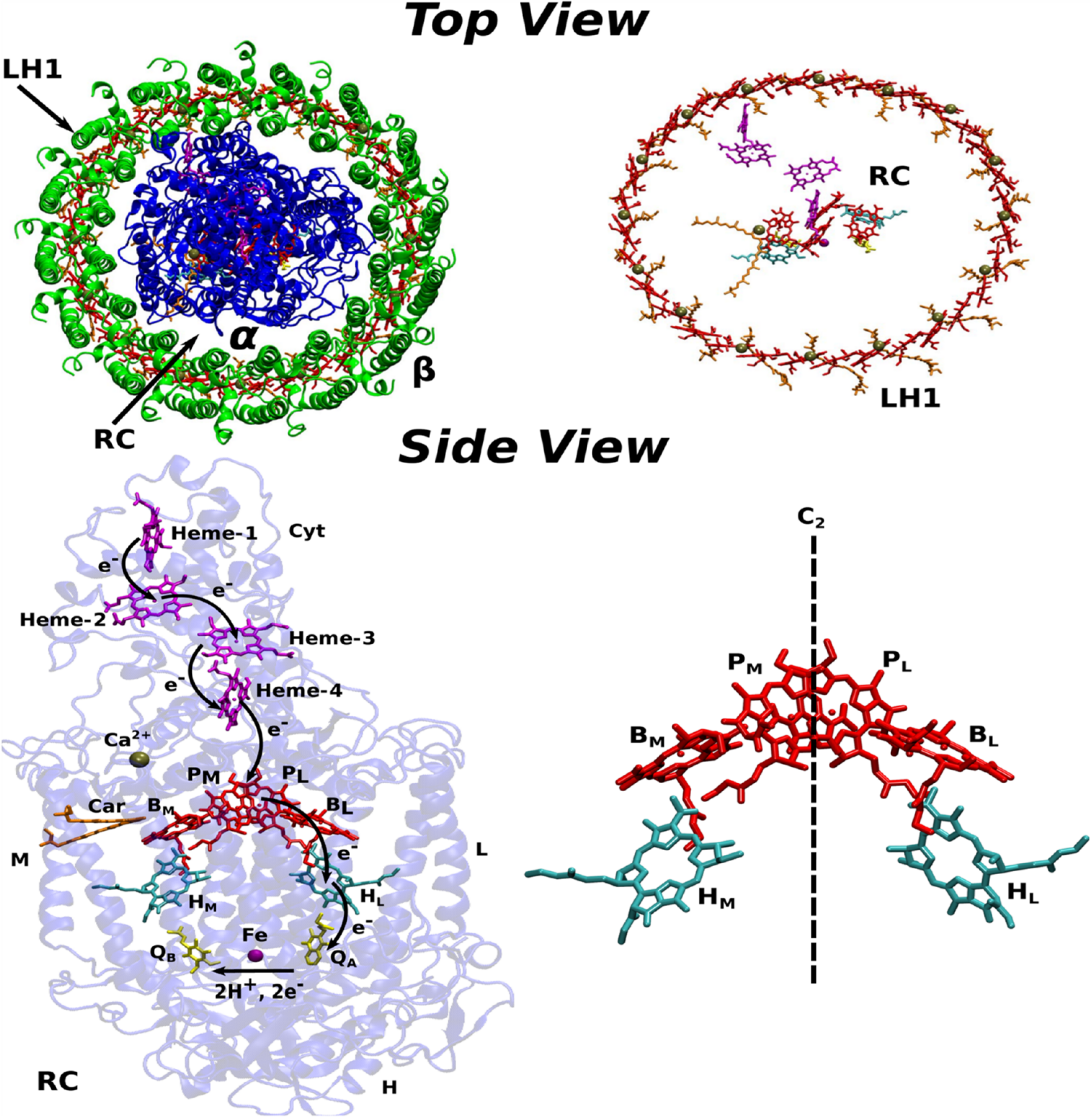
Top row: Top views of the LH1-RC complex from the purple bacterium *T. tepidum*,^12^ showing the RC (blue) enclosed by an elliptical LH1 ring (green) containing α (inner) and β (outer) polypeptides. Bottom row: Side view of the RC complex, including a sketch of the electron pathway through the pigment network in the active L-branch. BChls are shown in red, BPhs in cyan, Hemes in magenta, Cars in orange, and quinones in yellow color. In addition, only the BChl and BPh pigments within the PbRC are shown, highlighting the *C*_2_ symmetry.

Although extensive theoretical and experimental research has focused mainly on LH2 complexes,^17–20^ studies on coupled LH1 and RC complexes remain limited.^21,22^ Experimentally, Ma et al.^22^ identified a detrapping mechanism of energy transfer from the RC to the LH1 complex using 2DES, showing that calcium binding to the polypeptides of the LH1 ring modulates this process through effects on structure and spectroscopic properties. Moreover, recent studies show that, in *Thermochromatium tepidum*, uphill energy transfer from LH1 to the RC special pair is facilitated by thermal activation and a dedicated pathway formed by optimally oriented LH1 pigments.^23^ Furthermore, another study found that 61% of the red shift in the absorption spectrum of *Rhodospirillum rubrum* is due to contributions from CT states.^24^ However, the key questions about the PbRC complex are still unresolved, such as identifying the fundamental characteristics and precise location of the initial excited states, as understanding the properties and energy levels of CT states that enable efficient charge separation, and as uncovering the factors defining the electronic characteristics of the RC chromophores and the directionality of the electron transfer.

The validity of the long-held assumption that the charge separation is initiated at the RC due to its structural positioning and minimal interpigment distance has been questioned for a while already for various systems. Recent results in the theoretical analysis of photosystem II (PSII) in higher plants, utilizing time-dependent density functional theory (TD-DFT) within a quantum mechanics/molecular mechanics (QM/MM) framework, revealed a strikingly different behavior: the low-lying charge-transfer (CT) state was found not to be located at the special pair of the chlorophyll molecules.^25,26^ This finding, later confirmed by multispectral two-dimensional electronic-vibrational (2DEV) spectroscopy,^27,28^ underscores that the protein matrix exerts a very strong and almost exclusive control over the CT states in the pigment pairs, decisively shaping the charge separation.^25,26^ Nevertheless, although both multispectral studies^27,28^ consistently identify the same primary acceptor, they differ in their characterization of the donor. Fleming and co-workers^27^ reported a single ultrafast charge-separation pathway, whereas Ogilvie and co-workers,^28^ through 2DEV measurements combined with a continuum 2DES probe, uncover two distinct donor pathways operating on subpicosecond and picosecond time scales, respectively. Moreover, various studies have proposed models involving either a single or dual primary donor for the initial charge separation within PSII-RC complexes based on ultrafast experiments or TD-DFT calculations.^29–33^ Furthermore, recent parallel experimental^34^ and theoretical^35^ studies on heliobacteria RCs (HbRC) further undermine the belief that a low-lying CT state is present at the special pair. All of these studies suggest that primary charge separation in the reaction centers of various photosystems is unlikely to occur at the so-called special pairs. Although theoretical studies specifically addressing purple bacteria RCs are limited, previous experimental investigations on the RC from *Rhodobacter sphaeroides* also suggest that the initial charge separation may not take place at the special pair, though the exact location of the low-lying CT state in purple bacteria remains unclear.^36,37^ The proposed mechanisms for charge separation in these systems include transitions such as B_L_* → B_*L*_^+^H_*L*_^–^ or B_M_* → [P_*L*_P_*M*_]^+^B_*L*_^–^, where B_L_* and B_M_* represent excitonic states associated with the respective pigments. Moreover, a recent 2DES study positions the low-energy excited state of the special pair as an excitonic state [P_*L*_P_*M*_]*, suggesting a two-step charge separation pathway. This charge separation [P_*L*_P_*M*_]* → [P_*L*_P_*M*_]^+^H_*L*_^–^ progresses through an intermediate [P_*L*_P_*M*_]^+^B_*L*_^–^ in the active L-branch, a configuration observed in *Rhodobacter capsulatus*, where the active L- and inactive M-branches are distinct.^38^ Reinforcing this insight, a theoretical study using TD-DFT in a QM/MM framework, incorporating an implicit PCM model for the environment, revealed an ultrafast electron transfer from [P_*L*_P_*M*_]* to H_*L*_ via B_*L*_ in the active L-branch of PbRC in *R. sphaeroides*.^39^ In addition, this study comments that Tyr-M210 near the B_*L*_ pigment energetically stabilizes the [P_*L*_P_*M*_]^+^B_*L*_^–^ state and accelerates the electron transfer process. These studies were limited to an isolated, single-geometry crystal structure of the PbRC complex using an implicit solvent model, without conducting an in-depth investigation of the CT state present within the pigment pairs.

The focus in the present investigation is on analyzing a crystal structure of LH1-RC derived from the purple bacterium *T. tepidum*.^12^ The RC is surrounded by a set of 16 heterodimers, comprising LH1 αβ subunits, which together form a closed elliptical structure, as visually represented in Figure 1. Within the LH1 ring, a total of 32 BChl molecules and 16 spirilloxanthin carotenoids (Car) are symmetrically arranged. Furthermore, the periplasmic side of the LH1 ring contains Ca^2+^ ions, which play a crucial role in providing thermodynamic stability to the system. The RC is composed of cytochrome Cyt and three distinct protein subunits, namely *L*, *M*, and *H*. As mentioned previously, the *L* and *M* subunits consist of the special pair, i.e., P_*L*_ and P_*M*_, two associated BChls pigments termed B_*L*_ and B_*M*_, as well as one Car molecule. Moreover, it also contains two BPh, such as H_*L*_ and H_*M*_, as mentioned earlier. In addition, the Cyt unit binds four Heme groups responsible for transporting electrons to the special pairs. Furthermore, the RC contains two electron acceptors, namely menaquinone (MQ) and ubiquinone (UQ), which are, as mentioned earlier, referred to as Q_*A*_ and Q_*B*_, respectively. Moreover, in the crystal structure, a non-Heme iron atom is present in its oxidation state +3 situated between the two electron acceptors.^12^ The coordination of this iron atom together with nearby protein residues, specifically the glutamine (GLU) residue 234 and the histidine (HSD) residues 199, 219, 239, and 266 plays a crucial role in maintaining the structural stability of the RC and ensures an efficient functioning of the system.^40^

In this study we investigated the location of low-energy CT states and the initial charge separation within the BChl/BPh chromophore clusters in the reaction center, following excitation transfer from the LH1 complex, as well as the processes involved. To achieve this, we employed a multiscale approach that combines classical molecular dynamics (MD), QM/MM MD, and excited-state calculations using the TD-DFT and TD-DFTB methods. Interestingly, within the P_*L*_/P_*M*_ special pair, no low-energy CT state was detected. Instead, a low-lying CT state is present in the adjacent P_*L*_/B_*L*_ pair within the active L-branch. Upon further analysis, a dynamic interplay between locally excited (LE) states and pure CT states was evident throughout the simulation in this pair. These shifts between states possessing excitonic and CT characteristics suggest that charge separation is a complex, multifaceted process that occurs over various time scales to enable effective charge localization. Further examination indicates that the protein environment is critical in stabilizing CT states across different pigment pairs. Notably, a low-energy CT state was also observed between P_*M*_/B_*M*_ in the nonactive M-branch, exhibiting similar charge localization issues as the active branch. However, the direction of this CT state changes throughout the simulation, leading to slower or inactive charge separation in this branch. This low-energy CT state can be attributed to the symmetrical design of the active and inactive branches, with the asymmetry in the protein environment serving as the primary factor influencing the directional fluctuations in the respective CT states. Furthermore, when analyzing the three BChl molecules P_*M*_, P_*L*_ and B_*L*_ together, a low-energy CT state [P_*L*_P_*M*_]^+^B_*L*_^–^ was observed only with the protein environment, mainly dominated by P_*L*_/B_*L*_ interaction, consistent with prior experiments on PbRC.^16,38^ Finally, computationally demanding calculations including all BChl and BPh pigments in the active L-branch revealed a two-step charge dissociation pathway: [P_*L*_P_*M*_]* → [P_*L*_P_*M*_]^+^B_*L*_^–^ →[P_*L*_P_*M*_]^+^H_*L*_^–^ matching recent ultrafast experiment study^16,38^ but occurring exclusively within the protein environment as highlighted in the present study.

## 2 Results and Discussion

The key findings focus on the excitation energy landscape within the LH1 and RC complex, as well as the primary charge-separated state involving pigment pairs in the RC. These results were obtained using a multiscale approach that integrates classical MD simulations, QM/MM optimization and QM/MM MD followed by excited-state calculations employing TD-DFTB and TD-DFT methods. The results are detailed in the following subsections below.

### 2.1 Excitation Energy Distributions within the LH1 Complex

The quantum-chemical calculations started with a classically equilibrated system, focusing on the 32 BChl pigments in the LH1 ring of the LH1-RC complex to map the pathways of excitation energy transfer. Starting from a 200 ns-long classical MD trajectory, we computed the excited states in a QM/MM framework for the individual BChl across 20,000 equally spaced frames using the time-dependent long-range corrected density functional tight-binding method (TD-LC-DFTB) with the OB2 parameter set^41^ for which the range-separation parameter was set to 0.30 a_0_^–1^ with a_0_ being the Bohr radius. The TD-LC-DFTB approach is both computationally efficient and achieves near TD-LC-DFT accuracy, making it ideal for analyzing large protein-pigment LH complexes over extended trajectories without excessive computational costs.^20,42–44^ Moreover, average site energies based on the classical MD trajectories tend to be overestimated compared to those from DFTB/MM MD trajectories, though the overall trend remains the same, as shown in our previous study on the Fenna-Matthews-Olson complex.^42^ However, DFTB/MM MD simulations can only be run for a few nanoseconds, while here we intend to average over longer time spans to remove some of the artifacts resulting from averages over too short time ranges. Furthermore, we also performed the same calculations after removing the point charges representing the surrounding environment in order to assess the impact of conformational dynamics and the electrostatic influence of the protein environment on the excitation energies of individual pigment molecules in the LH1 complex. This approach has previously been applied to various light-harvesting complexes by our group^44–46^ and others^25,26,47–49^ to evaluate the effects of the electrostatic environment on the excited-state properties of pigment molecules.

For each BChl pigment, we calculated the first 10 excited states and extracted the lowest excited-state energy S_1_ also referred to as the Q_*y*_ site energy, in order to construct an excitation energy landscape within the LH1 ring. Figure 2 shows the distribution of the average Q_*y*_ excitation energies for the α and β pigments in the left panel, while the right panel presents the individual average values and fluctuations of each pigment throughout the trajectory, both with and without the surrounding environment. The excitation energy distribution, also known as the density of states, is represented by a histogram (bin width ∼0.013 eV) that has been smoothed using cubic spline fitting. The distributions show a Gaussian profile, frequently observed for pigment molecules in light-harvesting complexes.^43^ Although most BChl pigments experience similar fluctuations in excitation energies, certain pigments have slightly higher average energies, while others shift toward lower energies. The minor differences in the average site energies in Figure 2 result from environmental sampling effects. However, examining the average density of state for α and β pigments reveals nearly identical distributions. This similarity is expected, given that all BChl pigments linked to the α and β polypeptides are symmetrically arranged and exposed to very similar electrostatic environments within the LH1 ring. This finding is corroborated by the calculated average excitation energies without the environment, also displayed in Figure 2. These energies are slightly red-shifted relative to those determined within the environment via the QM/MM framework. Nevertheless, the energy variations between the individual pigments are negligible when the point charge environment is ignored. The conformational dynamics of the protein environment cause variations in the QM/MM energy profile, which is likely insufficiently sampled due to computational restrictions, leading to small differences in the site energies between the pigments. Although the excitation energies computed both with and without the environment are higher than the Q_*y*_ energies of individual BChl molecules in organic solvent, the magnitude of the energy shift between the two scenarios aligns well with findings from our previous research.^20^

**Figure 2:**
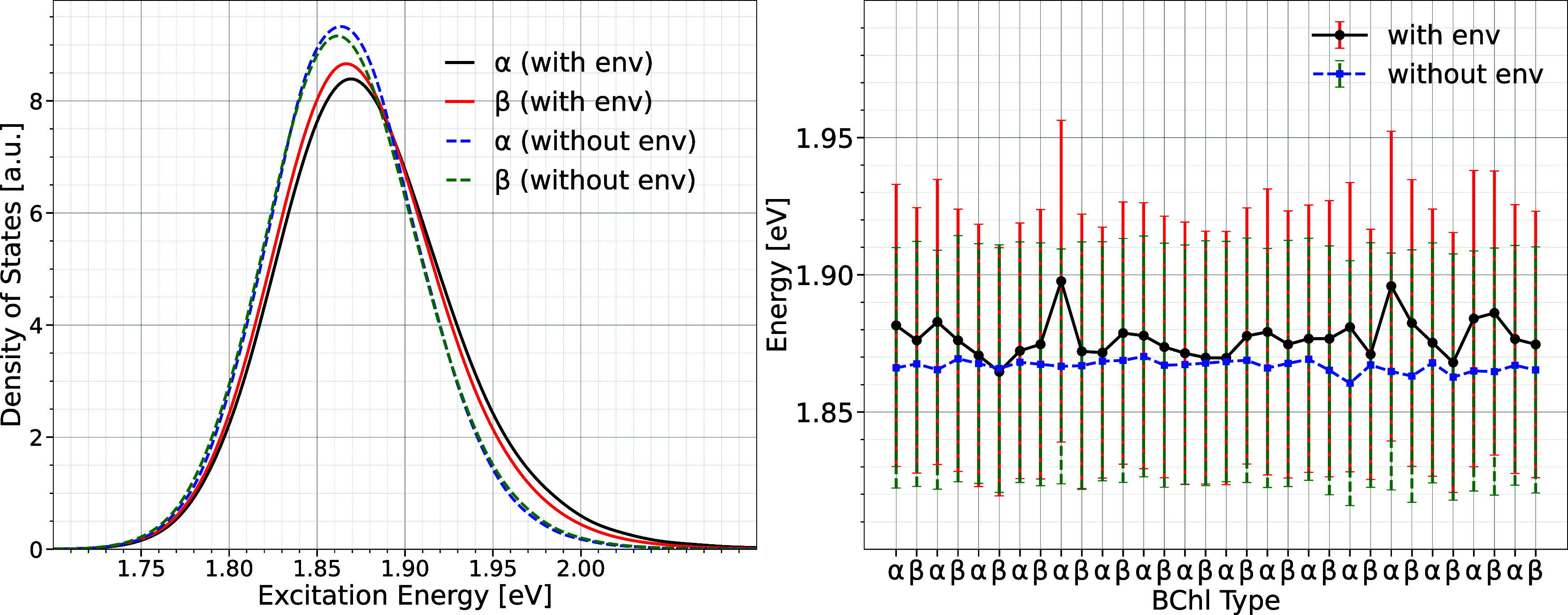
Left panel presents the average excitation energy distributions of the α and β BChl pigments in the LH1 ring, computed over a 200 ns classical MD trajectory using the QM/MM framework, both in the presence (solid lines) and absence (dashed lines) of the surrounding environment. The right panel depicts the mean excitation energies and standard deviations of the individual α and β pigments.

### 2.2 Excitation Energy Pathways and Primary Charge Dissociation in the RC

In the following step, we analyzed the excitation energy landscape within the RC complex, focusing on the four BChl and two BPh pigments. Again, we used the same 20,000 equally spaced frames from the 200 ns-long classical MD trajectory of the LH1-RC complex to extract the Q_*y*_ site energies of the pigment molecules at the TD-LC-DFTB level within the QM/MM framework. Similarly, the distribution and average excitation energy for each pigment were determined in the same way as for the LH1 ring. The associated distributions of the excitation energies, i.e., density of states, along with the corresponding average energies and fluctuations along the trajectory are shown in Figure 3. Moreover, Figure 3 indicates that the P_*L*_ and P_*M*_ molecules of the special pair have the highest and second-highest average site energies within the BChl network, suggesting that the highest excitonic state is likely localized within the special pair.

**Figure 3:**
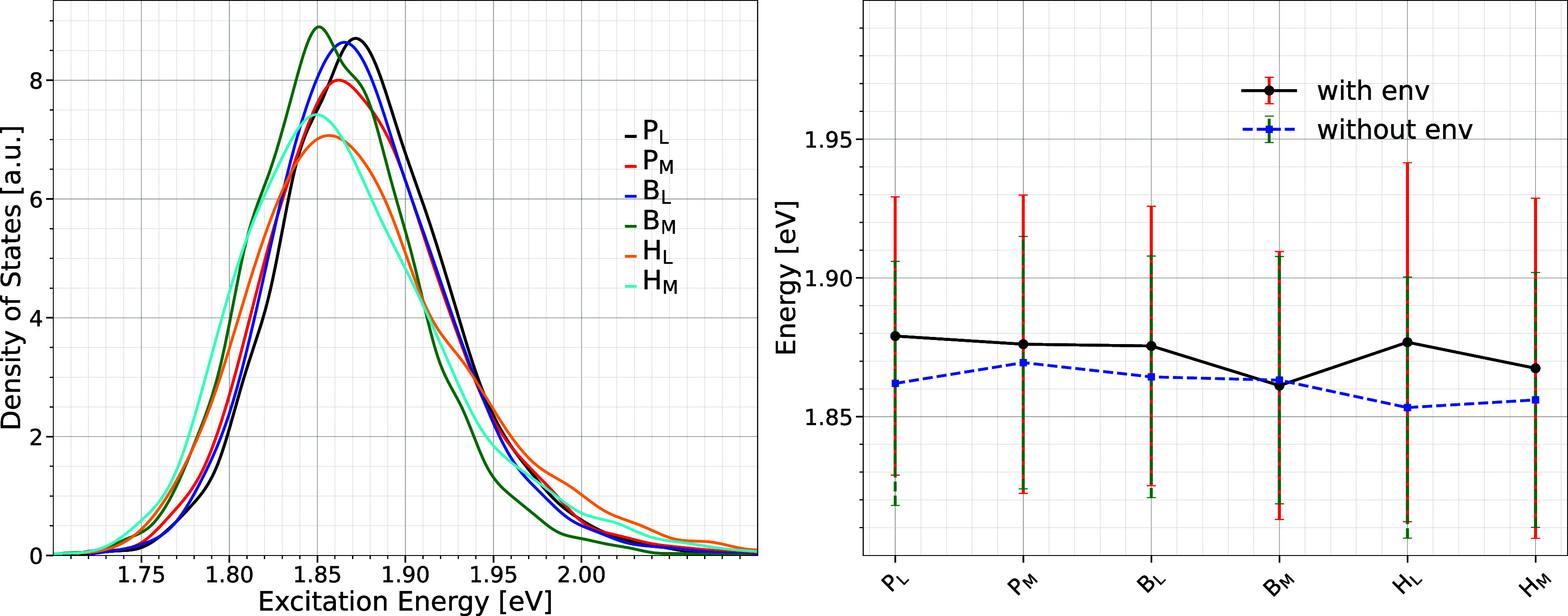
Same as in Figure 2 but for the pigments in the RC. In the left panel, the energy distributions are shown only for the case when the environment is taken into account.

Furthermore, the special pair pigments have energy levels that are larger compared to those of the majority of pigments within the LH1 ring. Specifically, the average excitation energy for the special pair P_*L*_ and P_*M*_ of 1.878 eV is either higher or comparable to the mean excitation energies of the LH1 ring with 1.877 eV. This finding reinforces the concept of an uphill energy gradient from the LH1 ring toward the RC, as evidenced earlier in an experimental study for the present system.^23^ Moreover, the high excitation energies of the special pair pigments within the RC align with previous findings using the semiempirical ZINDO/S-CIS (Zerners intermediate neglect of differential orbital with parameters for spectroscopic properties combined with the configuration interaction singles) method along with classical MD trajectories for the PbRC complex, where the highest and lowest excitonic states were identified on the special pair.^50^ In addition, we also found that B_*M*_ in the inactive branch has the lowest site energy in the pigment network of the RC. This suggests that this pigment may act as an energy sink to prevent excitons from propagating along the inactive branch and thus ensures an unidirectional energy transfer through the active L-branch. Although the active L- and the inactive M branches are symmetric in their pigment structure, the site energies of the individual pigments differ due to the asymmetric electrostatic environment of the protein, as can be seen in Figure 3. However, calculations without environment, as shown by the average site energies in the right panel of Figure 3, indicate that similar types of pigments have quite similar energies while their conformational space might be slightly different due to different binding geometries.

Furthermore, we have also performed TD-DFT calculations using the range-separation functional ωB97X together with the Def2-TZVP basis set to determine the excitation energies of each pigment within the RC in a QM/MM framework. Due to the computational cost of these calculations, they cannot be performed along the trajectory but on structures optimized in the QM/MM fashion using DFT at the PBE/DZVP-MOLOPT-GTH level of theory, together with the D3 dispersion correction. Additional details can be found in the Section 4, also for tuning the range-separation parameter ω. This optimally tuned ωB97X functional, which has also been used to analyze the excited-state charge transfer profile as described in the subsequent section, was used here to determine the excitation energies. Table 1 details the excitation energy results for both cases, the results with the default range-separation parameter default ω = 0.30 and the optimally tuned one, i.e., ω = 0.126. Moreover, we performed the calculation once with and once without the point charges of the environment. The shifts due to including the external point charges are in Table 1 as well.

**Table 1:** Q_*y*_ Excitation Energies (in eV) of the Individual BChl and BPh Pigments in the RC of the LH1-RC Complex with (QM/MM) and without (QM) Protein Environment Based on QM/MM Optimized Geometries^a^

	QM/MM		QM		shift	
pigments	ω = 0.30	ω = 0.126	ω = 0.30	ω = 0.126	ω = 0.30	ω = 0.126
P_*L*_	1.912	2.024	1.877	2.011	+0.035	+0.013
P_*M*_	1.833	2.000	1.838	2.011	–0.005	–0.011
B_*L*_	1.869	2.019	1.861	2.016	+0.008	+0.003
B_*M*_	1.826	1.995	1.849	2.014	–0.023	–0.019
H_*L*_	2.035	2.066	1.956	2.050	+0.079	+0.016
H_*M*_	1.900	2.029	1.885	2.025	+0.015	+0.004

a Moreover, the associated shift is also listed. The site energy calculations were performed using the range-separated DFT functional ωB97X with the Def2-TZVP basis set once using the default ω-value, i.e, ω = 0.30, and once using the tuned range-separation parameter of ω = 0.126.

TD-DFT calculations using both the default and optimally tuned ωB97X functional reveal that the pigment P_*L*_ exhibits the highest site energy, while B_*M*_ has the lowest one, consistent with the results from the TD-LC-DFTB calculations along the classical MD trajectory as presented earlier. In addition, the range of site energy shifts remains comparable between the default and tuned parameters for the BChl and BPh pigments. In the gas phase, i.e., without the environmental point charges, the excitation energies of the BChl molecules are quite similar, while the BPh molecules exhibit slightly higher values due to structural differences, in particular the absence of the Mg^2+^ ion at their center. However, within the QM/MM framework, the site energies of the BPh molecules fall within the same range as those of the BChl molecules, consistent with the findings from the TD-LC-DFTB calculations along the classical MD trajectory. Overall, in the active L-branch, the pigment molecules undergo a blue shift in the excitation energy, whereas a red shift is observed in the inactive M-branch. This behavior can likely be attributed to the asymmetry in the surrounding protein environment of the individual pigments, despite the structural *C*_*2*_ symmetry of the L and M branches. Notably, the extent of the shifts based on calculations with and without the point charges is consistent for each pigment in both branches, regardless of the environmental asymmetry.

#### 2.2.1 Excitation Profiles of the BChl Pairs in the RC

Next, we focused on various pairs of BChl pigment molecules in the active and the inactive branches including the special pair of the BChl molecules i.e., P_*L*_/P_*M*_ in order to identify energetically low lying or the even the lowest lying CT state in the RC. Other pigment pairs are considered such as P_*L*_/B_*L*_ and P_*M*_/B_*M*_ respectively. In the first section, we have carried out a natural transition orbital (NTO) analysis on the pigment dimer, followed by a density difference analysis. However, in the later section, we have expanded the density difference calculations, including groups of three and four pigments at a time. Thus, to begin, we optimized the ground state of these pairs using a QM/MM framework based on DFT calculations at the PBE-D3/DZVP-MOLOPT-GTH level and then performed TD-DFT calculations within the same setup based on the ωB97X/Def2-TZVP level of theory using the optimally tuned ω value. The structure obtained after classical equilibration served as the starting point for CT calculations of the pigment pairs. More information on these calculations can be found in the Section 4, and details of the various types of calculations performed in this study are listed in the SI. Because the CT states of the BChl dimers are optically dark (also described as polaron pairs in solid state physics), we identified them by evaluating the oscillatory strengths and transition dipole moments of the excited states. Additionally, we used an NTO analysis and a visualization of the difference densities. These are standard techniques to characterize excited CT states. Moreover, these analyses were performed both in the presence and absence of the environmental charges to assess the effect of the protein environment on the CT states (see the SI for properties of the ten excited states for different pigment pairs). After completing the TD-DFT calculations, we visualized the NTOs for each pair as depicted in Figure 4. In the case of a pure CT state, the relative contribution *n* in the NTO calculations typically lies in the range from 0.94 to 1.0, indicating that the excitation is dominated by a single hole-particle pair. These states usually exhibit very low oscillator strengths with values between 0 and 0.01 because of their dark state character. For a CT state, the hole localized on the Highest Occupied Molecular Orbital (HOMO) is primarily confined to the donor, while the electron localized on the Lowest Unoccupied Molecular Orbital (LUMO) is located at the acceptor. This separation of the charge density between the donor and the acceptor characterizes the charge-transfer nature of the excitation. If the contribution *n* of a specific NTO is higher than 0.90 and the corresponding oscillatory strength has a higher value, the state can be characterized as a mixture between CT and excitonic state. In the present study, we focus, however, on pure CT states in the pigment pairs. Figure 4 shows the lowest excited states, i.e., the S_1_ state, in different BChl pairs with and without taking the environment into account. This representation surprisingly reveals that the lowest-lying state in the P_*L*_/B_*L*_ pair is a CT state with the hole residing on the P_*L*_ pigment and the electron on the B_*L*_ pigment, i.e., P_*L*_^+^B_*L*_^–^. This finding aligns with previous studies where the primary charge separation in the PbRC does not happen at the special pair P_*M*_/P_*L*_.^37,51–54^ For that pair, the S_1_ state is a mixed excitation (ME) state delocalized across both pigments P_*M*_ and P_*L*_. In addition, in the case of the P_*M*_/B_*M*_ pair on the inactive branch, the S_1_ state is characterized by a local excitation (LE), localized either on P_*M*_ or on B_*M*_. The excitation energy levels follow the order P_*M*_/B_*M*_ (1.994 eV) larger than P_*L*_/P_*M*_ (1.882 eV) larger than P_*L*_/B_*L*_ (1.656 eV) within the QM/MM setting supporting the charge separation along the active L-branch. This order also aligns with earlier experimental observations that identify the intermediate [P_*L*_P_*M*_]/B_*L*_ as the primary site of charge dissociation in PbRC across various bacterial species.^37,51–54^ Interestingly, our calculations show that when the environmental point charges (MM) are excluded, i.e., the QM/MM coupling is turned off in the excited state calculations, the CT state is no longer present in the P_*L*_/B_*L*_ pair. However, for the special pair P_*M*_/P_*L*_, the S_1_ state remains largely unchanged, retaining the same ME characteristics as within the point-charge environment. Similarly, for the P_*M*_/B_*M*_ pair, the S_1_ state shows no significant change in the gas phase compared to the protein environment. In fact, in the case of the P_*M*_/B_*M*_ pair, the excited-state properties of the ten lowest excited states are unaffected by the protein environment, as shown in Table S3 of the SI. Furthermore, the gas-phase energy and the local excitation of the P_*M*_/B_*M*_ pair resemble those of the P_*L*_/B_*L*_ pair in the active branch. These results indicate that the protein environment plays a critical role in stabilizing the low-lying CT state in the P_*L*_/B_*L*_ pair within the active branch. Additional calculations neglecting the QM/MM coupling in TD-DFT calculations are crucial for analyzing the influence of the electrostatic protein environment on CT states. A similar effect has been reported in the RC complex of higher plants, where the CT state was significantly stabilized by the protein environment within a QM/MM framework.^25,26^

**Figure 4:**
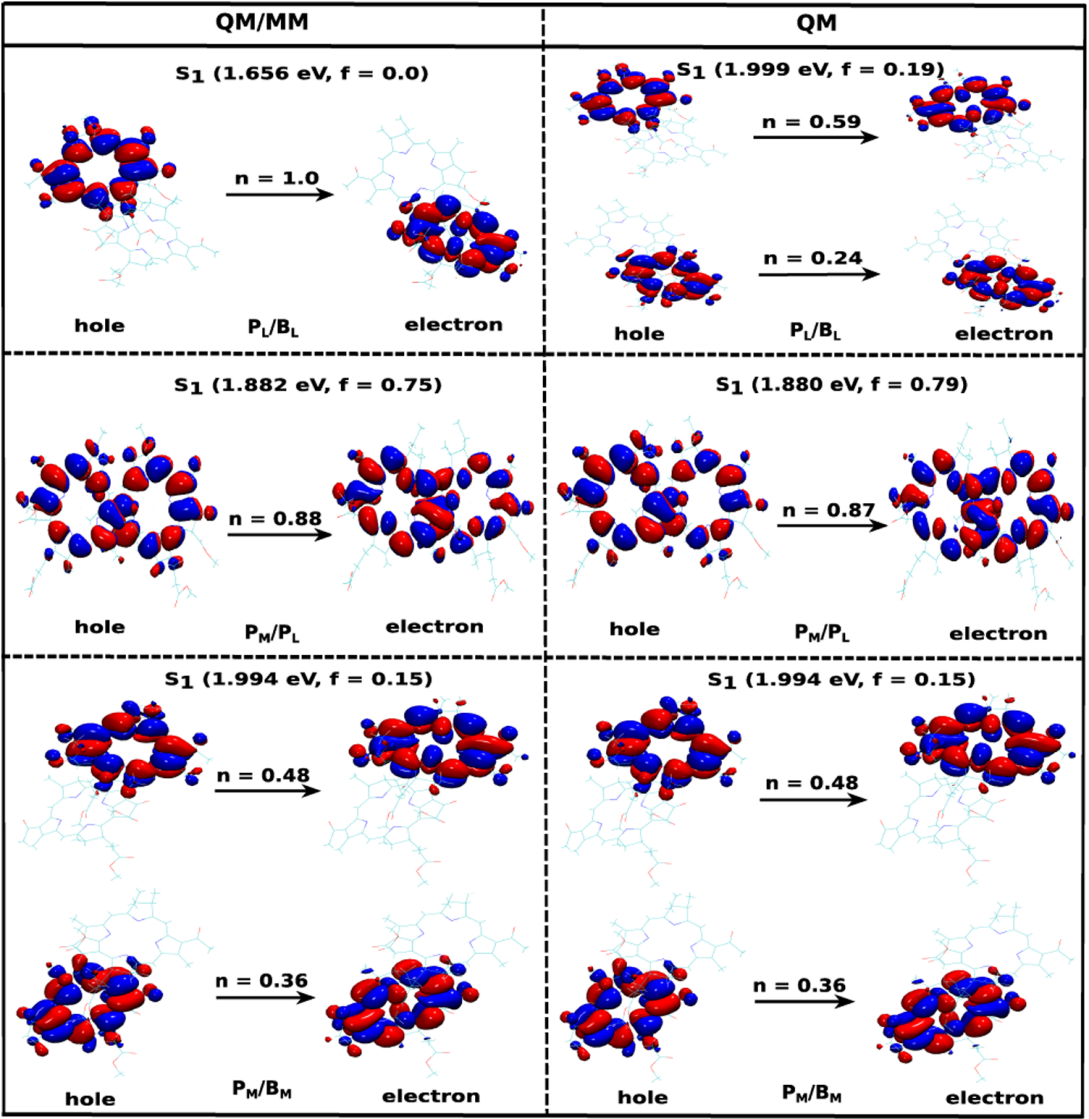
Natural transitions orbitals (NTOs) for different pigment pairs within the RC, including (QM/MM) and excluding (QM) the environmental influence on top of QM/MM optimized structures extracted after classical equilibration. The vertical excitation energies (in eV) and the oscillator strengths (f) are given in brackets for the lowest excited state, i.e., S_1_. In addition, the relative contribution *n* of a specific NTO to the given excitation is shown. The isosurface values for the wave function phases range from +1.28 × 10^–2^ (blue) to −1.28 × 10^–2^ (red) in atomic units.

Following the NTO analysis, the density difference between the ground and excited states was examined to identify the CT states. The density difference Δρ­(**r**) between the ground and excited states is defined as

1 $$\Delta \rho \left(r\right)=\rho_{\mathrm{excited}}\left(r\right)-\rho_{\mathrm{ground}}\left(r\right)$$

which can also be expressed in a simplified orbital-based approximation

2 $$\Delta \rho \left(r\right)\approx \sum_{\mathrm{ia}}c_{\mathrm{ia}}\left[\left|\psi_{a}\left(r\right){|}^{2}-\right|\psi_{i}\left(r\right){|}^{2}\right]$$

where ψ_*a*_(**r**) and ψ_*i*_(**r**) denote the molecular orbitals of the excited and ground states, respectively. In this context, Δρ­(**r**) > 0 indicates electron localization (electron gain), while Δρ­(**r**) < 0 corresponds to electron removal, i.e., hole formation. We performed the density difference analysis for the same BChl pairs as discussed above and the results are shown in Figure 5. Similar to the NTO analysis, the results clearly demonstrate that a *S*_1_ CT state is present only for the P_*L*_^+^B_*L*_^–^ pair, where the hole resides on the P_*L*_ and the electron on the B_*L*_ pigment. Notably, this CT state is formed exclusively in the presence of the environment, as can also be seen in Figure 5. Additionally, we identified energetically low-lying CT states for the special pairs P_*L*_/P_*M*_ as well as for the P_*M*_/B_*M*_ pair in the inactive branch. These states occur, however, either at higher energies or in the reverse direction, making them unfavorable for charge separation. Overall, this density difference analysis suggests that the density difference between the ground and excited state gives clear indications for CT states and thus will also be applied to identify the CT states in pigment pairs in the following sections.

**Figure 5:**
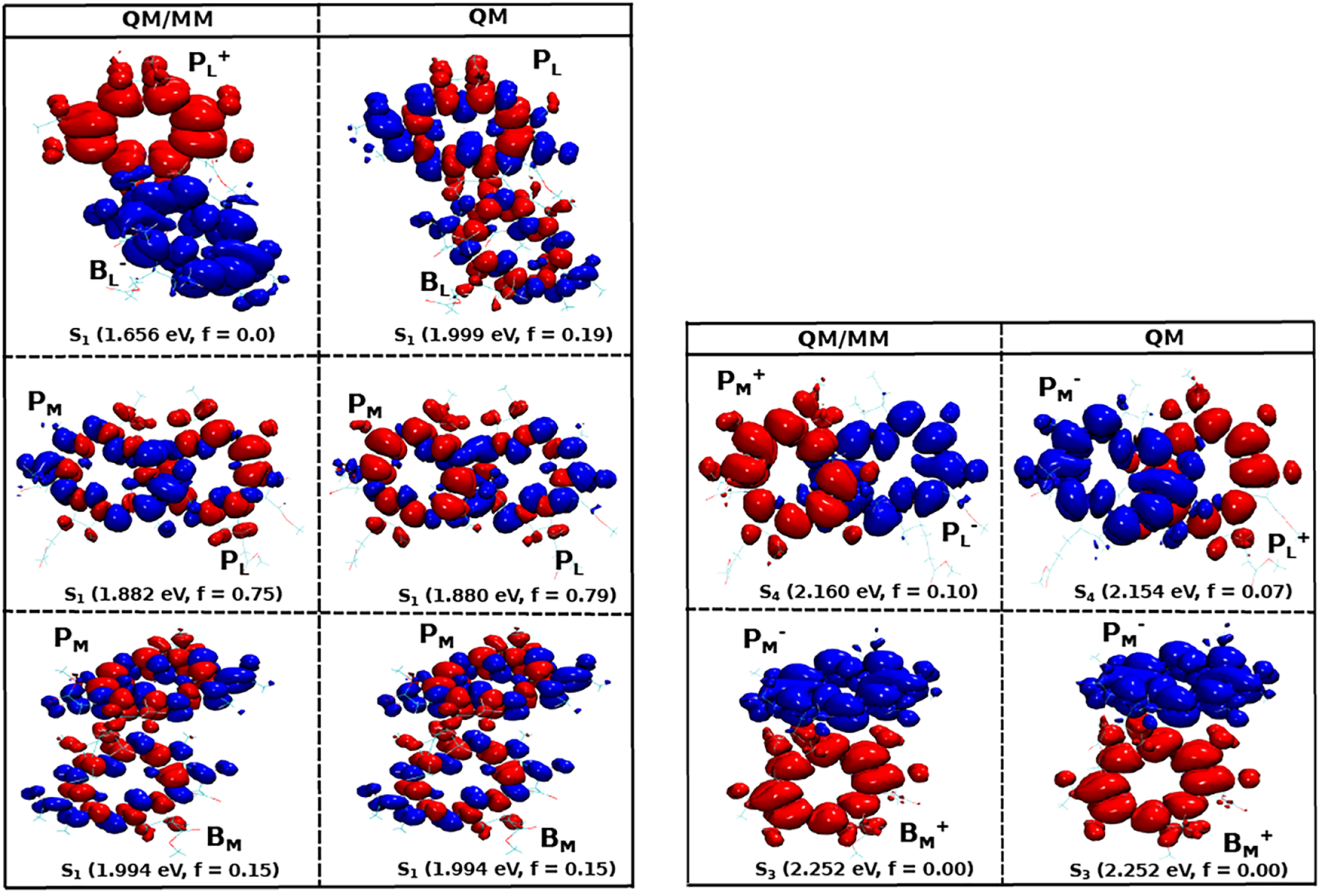
Similar to Figure 4 but for the density differences between the respective excited and the ground state. Also shown are the lowest CT states for the pairs P_*L*_/P_*M*_ and P_*M*_/B_*M*_. The isosurface values range between +1.0 × 10^–4^ and −1.0 × 10^–4^ for density gain (blue) and loss (red) in atomic units.

In the next step, we examined the density differences within BChl pairs for conformations extracted from the 200 ns MD trajectory with a 50 ns stride, i.e., the starting structures were at the time points 50, 100, 150, and 200 ns from the 200 ns classical MD trajectory. We employed the same QM/MM geometry optimization method, followed by TD-DFT computations for all these starting conformations. This approach provides a minimal sampling of the CT states for different pairs and their thermal fluctuations, while without the extra minimization step, we would run into geometry mismatch problems for excited state calculations on structures obtained from force field calculations. Again, we computed the first 10 excited states for all pigment pairs and tried to find low-energy CT states among them. Initially, we analyzed the first excited S_1_ state for the different BChl pigment pairs in the RC. The density differences are depicted in Figure 6. Interestingly, only the conformation obtained at 100 ns exhibits a CT state for the S_1_ excited state in the P_*L*_/B_*L*_ pair. Nonetheless, none of the S_1_ states in the special pair P_*M*_/P_*L*_ and P_*M*_/B_*M*_ has a clear CT character.

**Figure 6:**
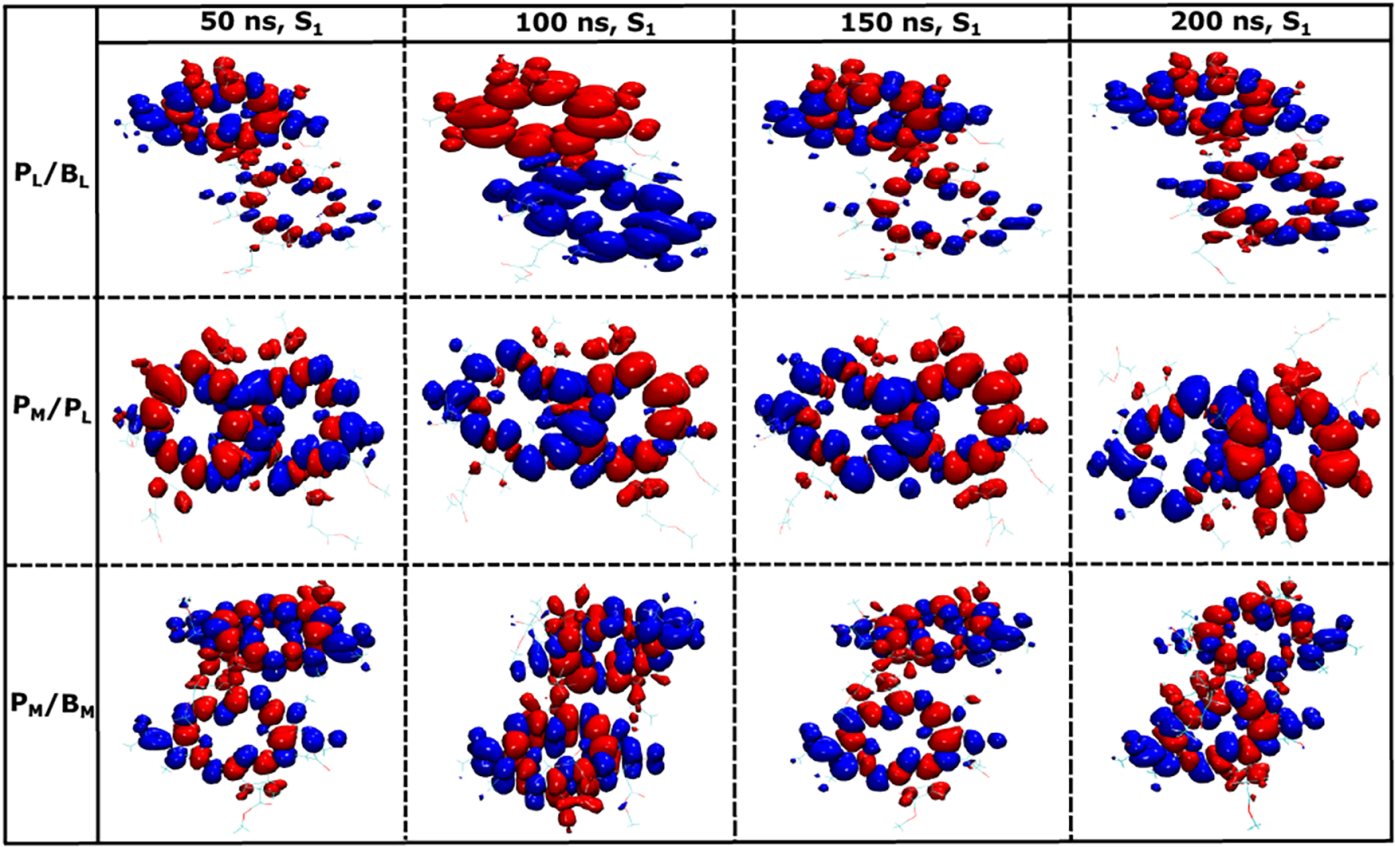
Similar to Figure 5 but for the S_1_ state based on QM/MM optimized structures for the snapshots extracted at 50, 100, 150, and 200 ns along a 200 ns classical MD trajectory.

Next, we analyze the S_1_ state and the low-lying CT states among the first 10 excited states along with their oscillatory strengths and NTO contributions as summarized in Table 2. From the table, it can be seen that in the case of the P_*L*_/B_*L*_ pair, CT states are found in the energy range of 1.987 to 2.289 eV corresponding to the states S_1_ to S_5_. In contrast, for the special pair P_*M*_/P_*L*_, the CT states occur at much higher energies above 2.512 eV, appearing in states S_7_ and S_8_. Interestingly, for the conformation at 50 ns, no CT state was observed for the special pair within the 10 lowest excited states. At the same time and for all starting conformations, the S_1_ state for the special pair exhibits a value for the NTO contribution exceeding 0.90, these reflect a mixed excitation (ME) character delocalized on the two pigments as shown in Figure 6. Furthermore, comparable ME states were identified earlier, as shown in Figure 4, with NTO contribution values of 0.88 and 0.87 for the first QM/MM optimized geometry following classical equilibration, both with and without point charges. For the P_*M*_/B_*M*_ pair in the inactive branch, the S_1_ state is not a CT state. However, CT states are observed between the states S_3_ and S_5_ within the energy range of 2.175 to 2.471 eV. In a further analysis, we demonstrate the existence of pure CT states in these pairs using the difference density analysis, as shown in Figure 7. This figure reveals that a low-energy CT consistently exists in the P_*L*_/B_*L*_ pair with the hole localized on P_*L*_ and the electron on B_*L*_, i.e., P_*L*_^+^B_*L*_^–^ for all snapshots (see upper panel in Figure 7). For the special pair P_*L*_/P_*M*_, however, the CT behavior is markedly different. Here, CT states can be observed only at high energies or with alternating directionality of the charge separation, not resulting in a consistent charge separation for different starting structures.

**Table 2:** Excitation Energies for the Lowest Excited State S_1_, for the Lowest CT State, and for the Higher Excited State S_10_ Together with the Respective Associated Oscillatory Strengths of the CT States and the NTO Contributions for Different Pigment Pairs Extracted at Different Times^a^

pair	time (ns)	S_1_ (eV)	CT (eV)	S_10_ (eV)	f (S_1_)	f (CT)	NTO (S_1_)	NTO (CT)
	50	1.992	2.277	3.197	0.27	0.01	LE(P_*L*_): 0.72	CT(P_*L*_^+^B_*L*_^–^): 0.94
	100	1.983	1.983	3.229	0.00	0.00	CT(P_*L*_^+^B_*L*_^–^): 1.0	CT(P_*L*_^+^B_*L*_^–^): 1.0
**P**_***L***_/**B**_***L***_	150	1.986	2.289	3.215	0.27	0.00	LE(P_*L*_): 0.72	CT(P_*L*_^+^B_*L*_^–^): 1.0
	200	2.000	2.237	3.273	0.13	0.00	LE(P_*L*_, B_*L*_): 0.45, 0.38	CT(P_*L*_^+^B_*L*_^–^): 1.0
	50	1.816	–	3.178	0.73	–	ME(P_*M*_/P_*M*_): 0.91	–
	100	1.775	2.707	3.175	0.59	0.00	ME(P_*M*_/P_*L*_): 0.93	CT(P_*M*_^+^P_*L*_^–^): 0.95
**P**_***M***_/**P**_***L***_	150	1.863	2.512	3.064	0.62	0.00	ME(P_*M*_/P_*L*_): 0.91	CT(P_*M*_^–^P_*L*_^+^): 0.96
	200	1.829	2.713	3.181	0.62	0.00	ME(P_*M*_/P_*L*_): 0.93	CT(P_*M*_^–^P_*L*_^+^): 0.97
	50	1.992	2.252	3.229	0.15	0.00	LE(P_*M*_, B_*M*_): 0.56, 28	CT(P_*M*_^+^B_*M*_^–^): 1.0
	100	1.980	2.175	3.195	0.14	0.00	LE(P_*M*_, B_*M*_): 0.50, 0.34	CT(P_*M*_^–^B_*M*_^+^): 1.0
**P**_***M***_/**B**_***M***_	150	1.992	2.187	3.198	0.18	0.00	LE(P_*M*_, B_*M*_): 0.48, 0.36	CT(P_*M*_^+^B_*M*_^–^): 1.0
	200	1.994	2.471	3.228	0.18	0.00	LE(P_*M*_, B_*M*_): 0.59, 0.25	CT(P_*M*_^–^B_*M*_^+^): 1.0

a LE denotes a local and ME a mixed excitation.

**Figure 7:**
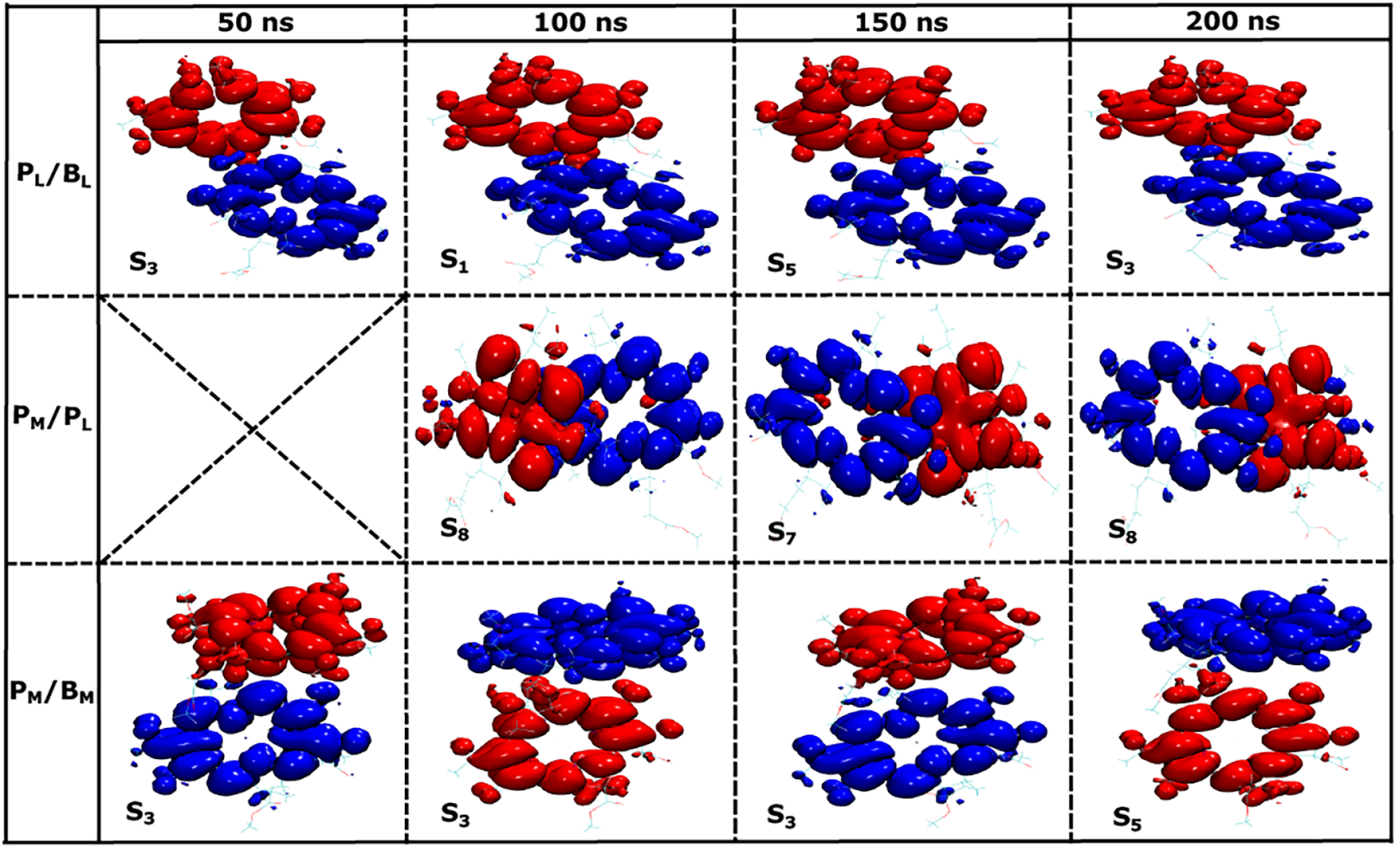
Similar to Figure 6 but for the lowest-lying CT states.

A fluctuating directionality of charge separation, as shown in the middle panel of Figure 7, is unlikely to result in efficient charge transfer on average. In the case of the P_*M*_/B_*M*_ pair within the inactive branch, the energy range is comparable to that of the active branch. However, also for this pair, the charge separation directionality undergoes frequent changes between P_*M*_^+^B_*M*_^–^ and P_*M*_^–^B_*M*_^+^ for different starting structures, disrupting any efficient charge separation (see lower panel in Figure 7). This irregularity likely contributes to the classification of that branch as inactive or slow, as the dynamic CT direction prevents a stable accumulation of charges necessary for functional operation. This analysis underscores the critical role of the CT stability and consistency of its directionality in determining the efficiency of the charge-transfer pathways.

To further investigate the effect of thermal fluctuations on the CT states, we performed computationally intensive 1 ps QM­(PBE-D3/DZVP-MOLOPT-GTH)/MM MD simulations. These simulations were initiated from the first QM/MM optimized structure after the classical equilibration, as well as from QM/MM optimized structures extracted at 50, 100, 150, and 200 ns. Frames were recorded every 10 fs, resulting in a total of 500 snapshots from all trajectories together for analysis. These frames were also subjected to TD-DFT calculations using the (ω = 0.126)­B97X functional to analyze CT states along the trajectory. We have again extracted the 10 energetically lowest excited states and further analyzed the lowest-energy CT state among them. These calculations were performed for the P_*L*_/B_*L*_ and P_*M*_/B_*M*_ pairs in both the active and the inactive branch. For the special pair, the TD-DFT calculations along the trajectory failed to converge due to problematic ground-state SCF cycles during the TD-DFT calculations (“geometry mismatch between ground and excited state approaches”), leading to artifacts in the excited-state energy values. This underscores the sensitivity of CT excited states to the strong coupling within the special pair and emphasizes the need for highly accurate methods. Optimal tuning of the range-separation parameter alone is likely insufficient, as is the quality of the geometry, given that the calculations consistently succeed only for QM/MM-optimized geometries. For the P_*L*_/B_*L*_ and the P_*M*_/B_*M*_ pairs, the distributions of the excitation energies was plotted similar to the ones in Figures 2 and 3 but with bin width ∼0.245 eV. They are based on 500 snapshots from five different starting structures and shown in Figure 8. For the P_*L*_/B_*L*_ pair, the CT state is observed to be within the range of the S_1_ to S_5_ states, whereas for the P_*M*_/B_*M*_ pair, it is confined to the range S_3_ to S_5_. While the average energies for the CT states are comparable in both cases, for the active branch, the distribution contains energy values below 1.8 eV, which is not the case for the inactive branch. Moreover, the CT state in the inactive branch shows directional fluctuations, as already discussed above and shown for the optimized structures in Figure 7. We would like to emphasize that extracting the directionality along a trajectory is challenging since the different starting structures already show a change in the direction in the MD structures which are 50 ns apart as shown in Figure 7 for the P_*M*_/B_*M*_ pair. Hence, the present analysis was performed for selected structures from the trajectory to extract only the excitation energies and their distributions.

**Figure 8:**
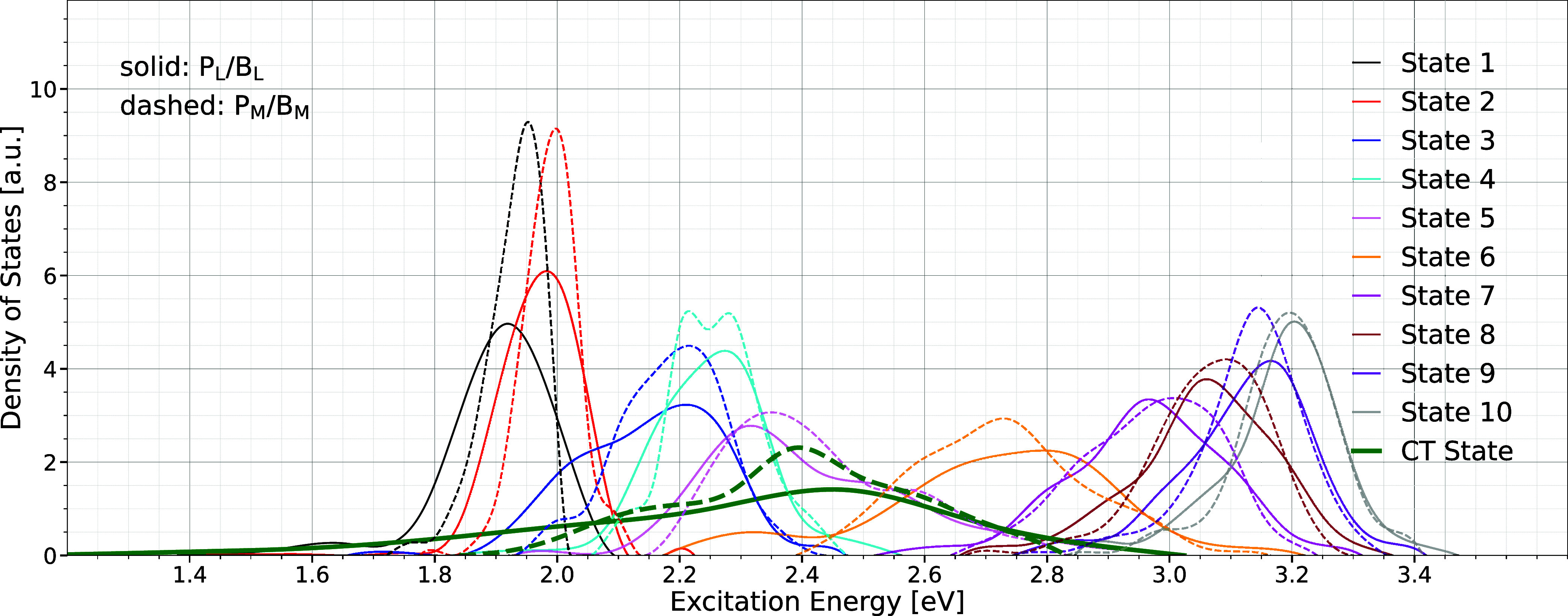
Energy distributions of the 10 lowest excited states are shown for the P_*L*_/B_*L*_ pair (solid lines) and the P_*M*_/B_*M*_ pair (dashed lines), based on 500 snapshots extracted from five independent QM/MM MD simulation sets. In addition, the lowest-energy CT state extracted from these 10 lowest excited states is shown as thick green lines. It becomes clear that the CT states for P_*L*_/B_*L*_ can be lower in energy than those for the P_*M*_/B_*M*_ pair.

#### 2.2.2 Role of HOMO and LUMO in Long-Range CT States

Based on the pairwise calculations conducted so far, we found that the low-lying CT state is localized on the P_*L*_/B_*L*_ pair rather than on the special pair P_*M*_/B_*M*_. Interestingly, although the Mg–Mg distance is larger in P_*L*_/B_*L*_ compared to P_*M*_/P_*L*_, a CT state still appears in the former. To better understand this behavior, we examined the HOMO and LUMO orbitals and their spatial overlap for both pairs. In a pure CT excitation, the dominant natural transition orbitals (NTOs) often resemble the canonical HOMO and LUMO. This means the electron is excited from the HOMO (hole NTO) to the LUMO (particle NTO), as observed for the P_*L*_/B_*L*_ pair. In contrast, for mixed or delocalized excitations such as those found in P_*M*_/B_*M*_, the NTOs can involve combinations of several occupied and virtual orbitals and may not correspond directly to the canonical HOMO and LUMO. Here we focus on the typical HOMO and LUMO orbitals for both pairs, as shown in Figure 9. In the P_*L*_/B_*L*_ dimer, the HOMO is localized on the donor (P_*L*_) and the LUMO on the acceptor (B_*L*_), consistent with a pure CT state. Because the HOMO and LUMO are on separate molecules, their product (HOMO × LUMO), which reflects the spatial separation of the electron and hole is effectively zero, further supporting strong CT character. In contrast, for the P_*M*_/B_*M*_ pair, the HOMO and LUMO are delocalized across both molecules, and their product is significant, indicating a more mixed excitation. This observation is consistent with the NTO analysis shown in Figure 4. These results suggest that the degree of localization of the frontier orbitals, particularly the HOMO and LUMO, is a key factor in enabling charge transfer in these molecular pairs.

**Figure 9:**
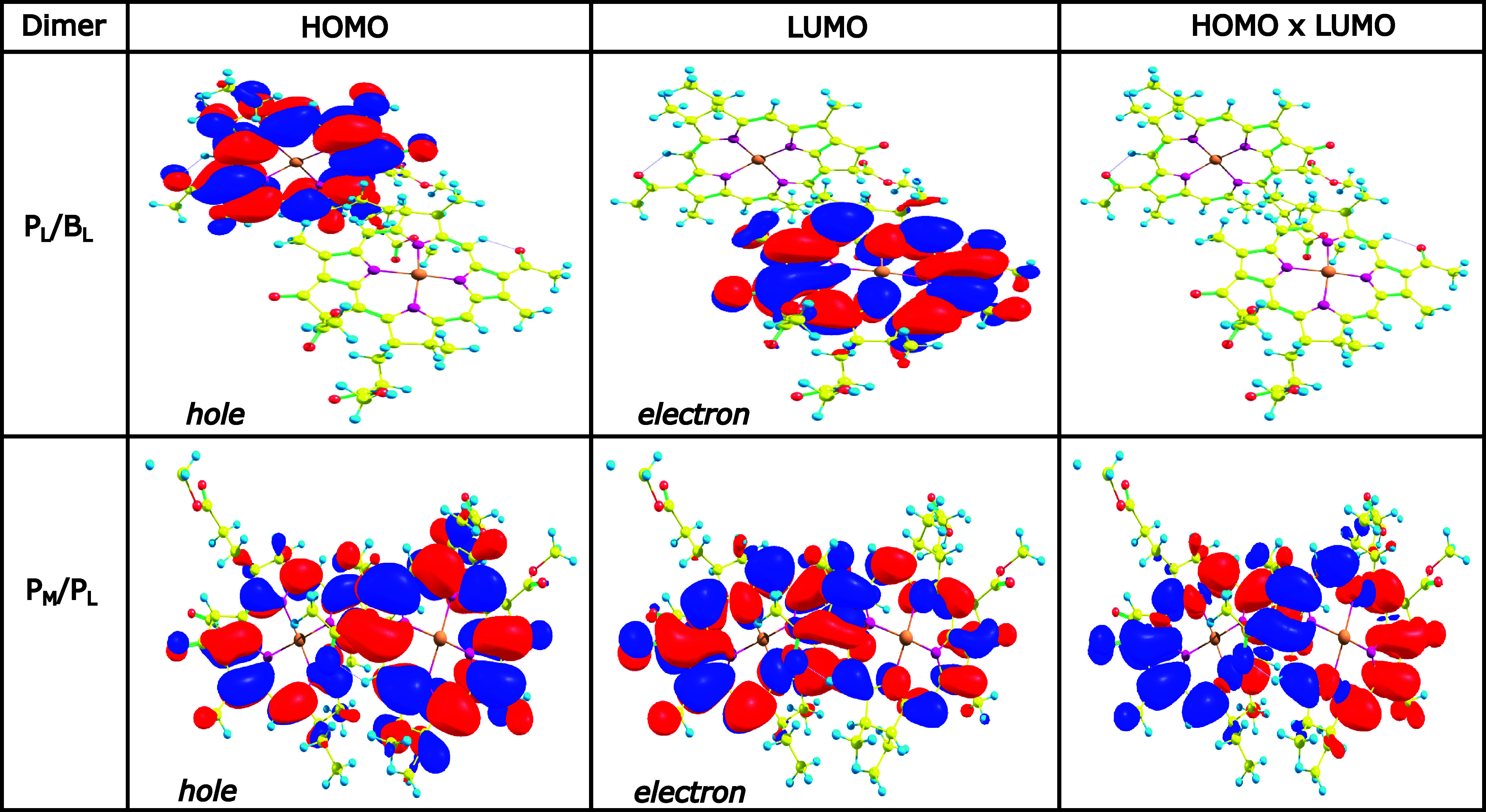
HOMO, LUMO and their product for P_*L*_/B_*L*_ and P_*M*_/P_*L*_ pairs depicted with isosurfaces of ±0.001 atomic units for HOMO/LUMO and ±0.0001 atomic units for the product.

#### 2.2.3 Excitation Profile of BChl Triples in the RC

Given the fact that experimental results also show that the initial position at which charge separation takes place is the P_*L*_/B_*L*_ pair, we further broadened our investigation. This involved an expansion of our analysis and the execution of calculations encompassing three BChl molecules in the QM region at a time, namely the special pair and its neighboring pigment from the active L-branch, i.e., [P_*L*_P_*M*_]/B_*L*_ and [P_*L*_P_*M*_]/B_*M*_ being present in the QM region for the QM/MM calculations. To this end, we started by optimizing the conformations of the three BChl pigments using the QM­(PBE-D3)/MM level of theory and subsequently carried out TD-DFT excited state calculations in the same QM/MM framework employing DFT calculations at the optimally tuned ωB97X/Def2-TZVP level of theory. Additionally, we repeated these TD-DFT calculations but omitting the environmental point charges (see SI for more details). The density difference for the S_1_ state was further analyzed and is shown in the upper half of Figure 10. The density difference analysis indicates that the S_1_ state is a pure CT state [P_*M*_P_*L*_]^+^B_*L*_^–^ with the electron localized at B_*L*_^–^, corroborating the aforementioned calculations for the P_*L*_^+^B_*L*_^–^ pair. Notably, when the environment is excluded, this CT state vanishes, highlighting its dependence on the specific environmental context. In addition, we conducted a set of calculations for the special pair and a pigment from the inactive M-branch, i.e., for [P_*L*_P_*M*_]/B_*M*_. The electron density difference of the S_1_ state is depicted in the lower half of Figure 10. Our results show that the S_1_ state of these three pigments in the inactive branch corresponds to an excitonic state that has the ME character in the P_*M*_P_*L*_ pair, both in the presence and absence of the environment, while B_*M*_ does not contribute significantly to this state. Importantly, this state has a higher energy than the corresponding one involving the active branch, underscoring the role of the M-branch as an inactive pathway for charge transfer on a slower time scale. However, because of the computationally demanding nature of the calculations, it is not feasible to examine the range of energies or the directionality of the CT state in the inactive branch containing three pigments across different snapshots or trajectories. However, it should be noted that in the case of two-pigment calculations, P_*M*_/B_*M*_ in the active branch exhibits the same range of energy as P_*L*_/B_*L*_ in the active branch. However, the directionality of the CT state fluctuates back and forth in the inactive branch, as discussed earlier.

**Figure 10:**
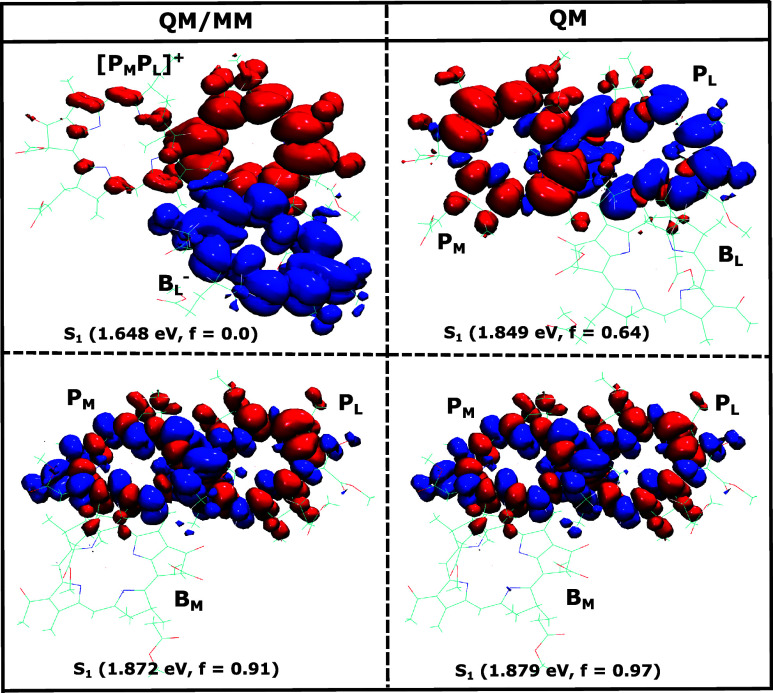
Density difference with and without QM/MM settings for the BChl triple including the special pair P_*L*_, P_*M*_ and one of the neighboring pigments, i.e., either B_*L*_ or B_*M*_ from the active L-branch (upper panel) or the inactive M-branch (lower panel).

#### 2.2.4 Excitation Profile of the Active L-Branch of the RC

Finally, we further extended our computations by performing numerically expensive QM/MM optimizations of the three BCL molecules (including the special pair) and one BPh in the active branch, followed by TD-DFT calculations, i.e., including P_*M*_, P_*L*_, B_*L*_ and H_*L*_ in the QM region (see SI for more details). The geometry obtained after classical equilibration was utilized as the initial structure for performing these calculations. We determined the density differences for low-energy CT states (see Figure 11) and identified that the S_1_ state, with an energy of 1.761 eV, shows a pure CT character and is denoted [P_*M*_P_*L*_]^+^B_*L*_^–^. Additionally, two other low-energy CT states were identified: S_6_ as [P_*M*_P_*L*_]^+^B_*L*_^–^ with an energy of 2.054 eV, and S_8_ as [P_*M*_P_*L*_]^+^H_*L*_^–^ with an energy of 2.153 eV. While these excited states correspond to S_6_ and S_8_, their energy values fall within the range of the previously mentioned low-lying CT state for the cases of including two or three BChl molecules as the QM region. These findings establish a mechanism of charge separation via a two-stage process: from excitonic [P_*L*_P_*M*_]* → [P_*L*_P_*M*_]^+^H_*L*_^–^, proceeding through an intermediate state [P_*L*_P_*M*_]^+^B_*L*_^–^, in agreement with recent experimental observations. Furthermore, we found that these CT states are strongly stabilized within the protein environment. However, as long as the environmental point charges were excluded, these CT states are either absent or formed in different pigment pairs, as illustrated in Figure 11. It is numerically not feasible, to perform many of these calculations, which include four pigments, for different snapshots or along a trajectory due to the numerically demanding geometry optimizations and QM/MM MD simulations required at the DFT level, followed by TD-DFT calculations. However, it is anticipated that low-lying CT states may fluctuate among other excited states that have at least a partial excitonic character, as demonstrated above for the two-pigment calculations.

**Figure 11:**
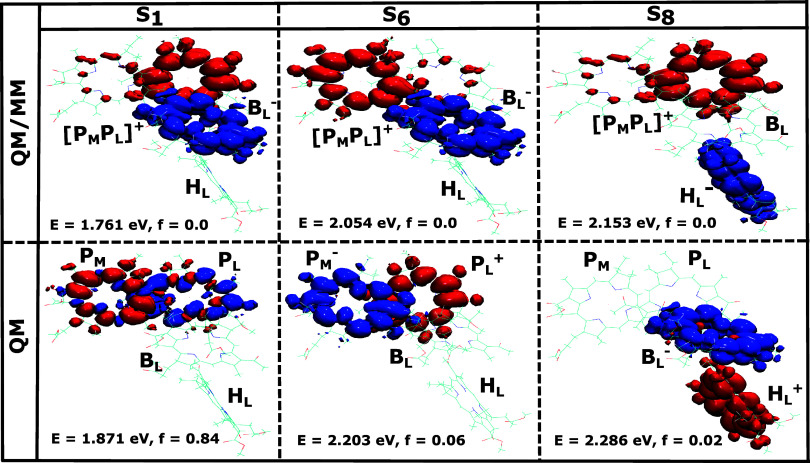
Same as Figure 10 but involving all BChl and BPh pigments in the active branch.

### 2.3 Absorption Spectra of the LH1-RC Complex

To validate the energy profile obtained from the TD-LC-DFTB calculations along the classical MD trajectory (see Figures 2 and 3), we computed the absorption spectra of the LH1-RC complex using an excitonic model (not including the charge transfer contributions). This approximation is reasonable because CT states, which are predominantly localized within the RC, mainly only cause a significant red shift of the absorption peak. The spectra were calculated using the second-order full cumulant expansion (FCE) method,^55^ where the excitonic Hamiltonian (see eq 3), transition dipole moments, and spectral density serve as input parameters. The FCE method has been successfully applied recently to the absorption spectrum calculation of light-harvesting complexes.^44,49,56,57^ The system Hamiltonian for describing the single exciton manifold within the Frenkel Hamiltonian model^58^ is given by

3 $$H=\sum_{m}E_{m}\left|m\right\rangle \left\langle m\right|+\sum_{m\neq n}V_{\mathrm{mn}}\left|n\right\rangle \left\langle m\right|$$

where the diagonal elements *E*_*m*_ denote the excitation energy, also referred to as the site energy of pigment *m*, and the off-diagonal elements *V*_*mn*_ represent the interpigment couplings. In this study, we have considered the so-called TrESP approach (transition charges from electrostatic potential)^59^ in which the coupling elements are given by

4 $$V_{\mathrm{mn}}^{\mathrm{TrESP}}=\frac{f}{4\pi \epsilon_{0}}\sum_{I,J}^{m,n}\frac{q_{I}^{T}\cdot q_{J}^{T}}{\left|r_{m}^{I}-r_{n}^{J}\right|}$$

where *q*_*I*_^*T*^ and *q*_*J*_^*T*^ are the transition charges of atoms *I* and *J*, respectively, and *f* is a screening factor that accounts for environmental effects on the excitonic coupling. Furthermore, in this study, we have employed a constant screening factor of 0.69, as recommended for light-harvesting complexes.^60^

Thus, to construct the excitonic Hamiltonian as shown in eq 3, the TrESP excitonic couplings were determined between all 32 BChls in LH1 as well as the 4 BChls and 2 BPhs in the RC complex using eq 4. Details on the calculation of the TrESP charges are provided in the Section 4. Since no spectral density data is currently available for the LH1-RC complex, we employed the experimental spectral density of the FMO complex^62^ employed in our previous study.^42^ No static disorder was included to further broaden the spectrum. The computed spectra were shifted by ∼ −4142 cm^–1^ toward lower frequencies to align with the experimental Q_*y*_ absorption peak position at 914 nm (see Figure 12). As noted above, this model does not include the CT contribution (for both LH1 and RC), however, a recent theoretical study showed that about 61% of the absorption spectral shift in the LH1 complex of *Rhodospirillum rubrum* purple bacteria arises from CT effects.^24^ In addition to the CT contribution, the overestimation of site energies by the TD-LC-DFTB method applied along the classical MD trajectory also contributes to the necessary spectral shift observed.^56,63^ However, overall, the simulated spectrum shows a good agreement with the experimental results, particularly in capturing the high-frequency vibrational features. This indicates that, despite the tendency of the TD-LC-DFTB approximation to overestimate excitation energies and the omission of CT contributions, which would otherwise lower the peak position, the general trend in the site energies remains appropriate for modeling the LH1-RC complex, as also done earlier for other bacterial and plant LH complexes.^43^

**Figure 12:**
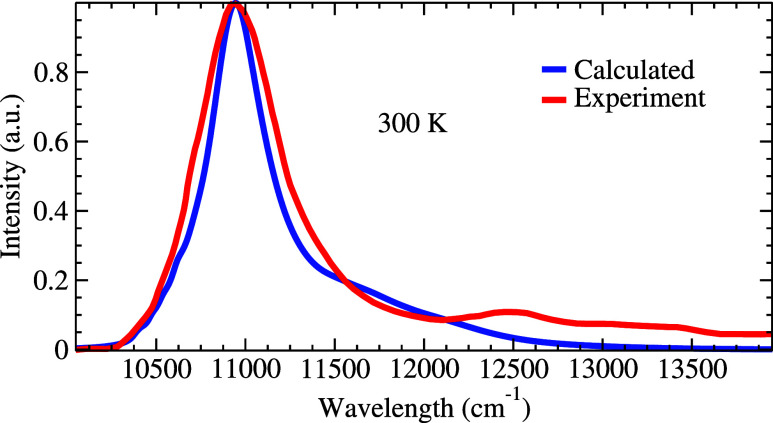
Calculated absorption spectrum of the LH1-RC complex at 300 K compared to an experimental one^61^ provided under an Open Access license. Reproduced from ref 61. Available under a CC-BY 4.0 license. Copyright © 2020 The Authors.

## 3 Conclusions and Outlook

The present study marks the first structure-based atomistic exploration of the entire LH1-RC complex of purple bacteria, providing valuable insights for future research into the overall exciton energy transfer between the LH1 ring and the enclosed RC complex. Additionally, an extended model can be developed by incorporating additional entities such as the involved quinones and/or BChl/BPh pigments of the inactive branch within the QM region, followed by the inclusion of protein residues. Furthermore, our study emphasizes the challenges of examining the charge separation process using a single geometry, whether derived from a crystal structure or from a QM/MM optimization. We addressed the issue that the charge transfer state can fluctuate significantly in energy and in its position among the excited states, depending on different initial geometries. An ideal solution would be to involve the inclusion of nonadiabatic techniques that incorporate the coupling between the excitation energy and the charge transfer in the Hamiltonian that determines of excited-state dynamics in an “on-the-fly” style. In such an approach, one would be able to study the exciton propagation followed by charge separation in RCs. However, these computations are computationally very expensive. In close collaboration with other research groups, we are exploring alternative strategies, including machine learning methods, that simulate the nonadiabatic dynamics using a surface-hopping approach. In this regard, simulations of exciton dynamics have shown promising results for the Fenna-Matthews-Olson (FMO) complex of green sulfur bacteria^64^ and the LH2 complex of purple bacteria.^65^ Furthermore, a more refined model that incorporates state-specific solvation^66^ or a polarizable force field for the environment^67^ can potentially provide additional stabilization to the charge transfer state, beyond what is observed in the present study based on nonpolarizable electrostatic embedding. However, such calculations are complex and often computationally expensive, particularly when performing excitation calculations that involve multiple pigments in the QM region. Additionally, we aim to further explore the connection to the heme-containing Cyt complex, which is another intriguing system involving electron transfer.^68^ We are particularly interested in understanding its role in the electron transfer to the RC complex. Therefore, the present study establishes the basis for a comprehensive examination of the energy and charge transfer mechanisms in the LH1-RC complex from an atomic perspective.

## 4 Material and Methods

The initial starting structure for the study is based on the structure 3WMM of the LH1-RC complex from the bacterium *T. tepidium* and has been obtained from the Protein Data Bank.^12^ A comparison to the similar structure 5Y5S^15^ is given in the SI. The protein was intricately embedded in a POPC bilayer with the help of the CHARMM-GUI Web server.^69^ Furthermore, we have inserted POPC lipids into the space between the LH1 ring and the RC complex, linking the two entities. We found that a maximum of 22 POPC molecules can be packed within the gap between the LH1 ring and the RC complex during equilibration. We want to note that the major thylakoid lipids embedded in natural LH1-RC complexes in *T. tepidum* are the negatively charged cardiolipin (CL), phosphatidylglycerol (PG), and the neutral phosphatidylethanolamine (PE).^15,70^ Accurately modeling such a realistic membrane for LH1-RC based on these lipids, however, is challenging due to difficulties in determining their proper composition and position. Using such a lipid mixture would, moreover, call for a proper sampling of various lipid positions to avoid artifacts due to positioning charged lipids close to pigments. POPC, a neutral phosphatidylcholine (PC) lipid, is a widely used and experimentally validated choice for MD simulations of light-harvesting membrane proteins. Such membranes closely mimic the hydrophobic thickness and surface area of thylakoid membranes and produce well-equilibrated structures.^5,71–73^ Since investigating protein–lipid interactions is beyond the scope of the present study, relying on the equilibrated structure within a POPC lipid bilayer is thus a reasonable compromise. Moreover, it is not expected that significant quantitative differences will occur due to the choice of the membrane lipids. The equilibration procedure for the system is explained in detail in the SI. The force field parameters for the cofactors were adopted from elsewhere.^50^ In the system setup and throughout the simulations, the iron (Fe) in the heme group was covalently bound to methionine (MET: resid 94, 130, 236) and histidine (HSD: resid 144, 156, 311, 251) with the bonding parameters taken from ref 74. Additionally, cysteine linkages were formed with the tail of the Heme group during the system preparation of the Cyt unit in the RC. This interaction helped to make the Heme group planar during equilibration. In the case of non-Heme iron, covalent bonding interactions were established with glutamine (GLU: residue 234) and histidine (HSD: residues 199, 219, 239, 266) using CHARMM-compatible parameters and partial charges from ref 40. This ensures the stability of the non-Heme iron and its surrounding environment throughout the simulation.

The systematic classical equilibration procedure of the system is described in the SI. The equilibrated structure was subjected to a 200 ns MD simulation, from which 20,000 frames were extracted with a 10 ps stride. These frames were then used to calculate the excitation energies for each pigment using the TD-LC-DFTB method as implemented in the DFTB+ code^75^ for the LH1 complex, including the RC complex. As a result, these calculations provide the excitation energy landscape within the LH1-RC complex, which is essential for determining the first step of the energy transfer process from the LH1 ring to the RC. Additionally, the 200 ns MD trajectory was used for the calculation of the excitonic couplings using the TrESP approach in order to construct the Hamiltonian together with the excitation energy. The TrESP charges were computed at the CAM-B3LYP/Def2-TZVP level using the ORCA software based on geometries optimized at the B3LYP/Def2-TZVP level, and the resulting transition densities were analyzed using the Multiwfn program^76^ to extract the transition charges. We also applied the electrostatic CHelpG fitting method for the TrESP charges, as implemented in Multiwfn. In addition, a scaling factor of 0.724 was applied to match the experimental transition dipole moment of BChl molecules. The same factor was used for the BPh molecules as well. Moreover, it is important to note that in this study, the semiempirical TD-LC-DFTB method was employed solely for calculating excitation energies along classical MD trajectories, rather than for analyzing CT states, as it has been benchmarked within our exciton model.^20,43^ While this method could potentially be used for CT states in pigment molecules, a proper benchmarking would need to be conducted, which falls outside the remit of this work.

In the charge transfer state calculations, the classically equilibrated system undergoes a QM/MM optimization, where the QM region is described using the DFT theory at the PBE/DZVP-MOLOPT-GTH level using GTH-PBE pseudopotentials and incorporating D3 dispersion correction. The Mg atom, however, was described with the DZVP-MOLOPT-SR-GTH-q10 basis set and the corresponding GTH-PBE-q10 pseudopotential. Moreover, the Gaussian Plane Waves (GPW) method was employed for the DFT as implemented in the CP2K quantum chemistry code, which was electrostatically coupled to the GROMACS simulation engine.^77–201^ The resulting QM/MM optimized structures were then used to calculate excited states within a QM/MM framework, utilizing the ωB97X long-range corrected (LC) DFT functional. The visualizations of the NTOs and the density differences for the CT states were performed using VMD,^78^ while the HOMO/LUMO orbitals were visualized with Chemcraft.^79^ Since the geometry from classical MD trajectories or crystallographic structures was insufficient for charge transfer calculations, QM/MM-optimized structures had to be determined. Additionally, even for minimal sampling, multiple geometries are necessary. To this end, we extracted snapshots from a 200 ns MD trajectory with a 50 ns stride, followed by QM/MM optimization and TD-DFT calculations. Furthermore, we conducted computationally demanding QM­(PBE-D3)/MM MD simulations of 1 ps length and extracted 500 frames for both the active and inactive branches, considering two BChl pigments at a time, which were subjected to further TD-DFT calculations. Details of the different types of computational calculations performed in this study are provided in the SI. The TD-DFT calculations for the QM/MM-optimized structures and along QM/MM MD trajectories in the RC were performed using the ORCA quantum chemistry code.^80^ To this end, we employed the Tamm–Dancoff approximation (TDA)^81^ and the Resolution of Identity (RI) approximations to speed up the TD-LC-DFT calculations. This was achieved by using the RIJCOSX approach^82^ for the Coulomb integral and the Hartree–Fock exchange, along with the Def2/J auxiliary basis set, as implemented in the ORCA program.^80^ Moreover, we employed the TightSCF convergence criterion, which imposes an energy change threshold of 1.0 × 10^–8^ au during the TD-LC-DFT calculations. Throughout the analysis, TDA-DFT calculations are referred to as TD-DFT. Moreover, to perform charge transfer calculations, it is essential to optimally tune the LC-DFT functional to comply with Koopman’s theorem. Since the default range-separation parameter ω = 0.30 in ωB97X does not accurately capture this aspect, we adjusted the parameter to better match the ionization potential (IP) and electron affinity (EA) of the BChl and BPh molecules, aligning them with the HOMO and LUMO energy levels. To this end, the range-separation parameter ω was fine-tuned to a value of 0.126 a_0_^–1^ with a_0_ denoting the Bohr radius. This adjustment has been recommended in particular for CT state calculations of BChl pigments^35,83^ as the default parameter in the DFT functional can lead to artifacts in large molecular systems such as BChl dimers, as shown in Table 3. In this table, the calculated ionization potential (IP) and electron affinity (EA) values for the BChl and BPh molecules are listed, comparing them with the ϵ^HOMO^ and ϵ^LUMO^ values for the tuned and default range separation parameters. The gas-phase geometries of BChl and BPh were optimized at the PBE/DZVP-MOLOPT-GTH level with the D3 correction using the CP2K code.^201^ These optimized geometries were then used for TD-DFT calculations with both the default (ω = 0.30 a_0_^–1^) and the optimally tuned (ω = 0.126 a_0_^–1^) ωB97X functional, along with the Def2-TZVP basis set as implemented in the ORCA quantum chemistry code. Moreover, we again utilized the TDA and RI approximation to speed up the calculations, as mentioned earlier. The formulas for calculating the IP and EA values are as follows: IP­(*n*) = *E*(*n* – 1) – *E*(*n*) and *EA*(*n*) = *E*(*n*) – *E*(*n* + 1), where *n* represents the total number of electrons in the respective molecule. Furthermore, it is worth noting that ideally the optical tuning should be performed within a QM/MM framework. However, this approach requires individual tuning for each pigment due to their distinct electrostatic environments, making the calculations tedious and computationally demanding when multiple pigments are considered for the CT state analysis. Therefore, for simplicity, the tuning was performed in the gas phase for the isolated pigment molecule, as also done in earlier studies.^19,35^

**Table 3:** Optical Tuning of the Range-Separation Parameter (ω in Units of a_0_^–1^) in the ωB97X Functional^a^

	ω = 0.30		ω = 0.126	
	BChl	BPh	BChl	BPh
ϵ^HOMO^(*n*)	–6.44	–6.52	**–5.80**	**–5.86**
IP(*n*)	5.89	5.97	**5.81**	**5.88**
ϵ^LUMO^(*n*)	–1.97	–1.85	–1.94	–1.83
EA(*n*)	2.42	2.32	1.89	1.81

a All values for *ϵ* ^HOMO^, IP, EA, and *ϵ* ^LUMO^ are given in eV. The *ϵ* ^HOMO^ and IP values for the tuned ω are shown in boldface.

## Supplementary Material



## Data Availability

The data that support the findings of this study are available from the corresponding author upon reasonable request. Additionally, the QM/MM-optimized geometries, point charges, QM/MM trajectories and TrESP charges have been deposited in Zenodo (https://doi.org/10.5281/zenodo.16938359).

## Notes

Equilibration procedures of the system, summary of computational details, structural comparison between 3WMM and 5Y5S and excitation profiles for various sets of pigment molecules (PDF)

## Notes

The authors declare that the present research has been mainly produced with publicly available software, as also detailed in the Section 4.
