# Supplementary material for: Multiscale Simulation of Primary Charge Separation Mechanisms in an LH1-RC Complex
Source: JACS Au. 2025 Oct 23;5(11):5609–25. doi: 10.1021/jacsau.5c01095 (PMC12648296; doi:10.1021/jacsau.5c01095)
Supplement: Supplementary file 1 [file au5c01095_si_001.pdf]

SUPPLEMENTAL INFORMATION
Multiscale Simulation of Primary Charge Separation Mechanisms in an LH1-RC Complex

Sayan Maity*,†,‡ and Ulrich Kleinekathöfer‡

†*Department of Physics and Astronomy and Thomas Young Centre, University College London, London WC1E 6BT, U.K.*

‡*School of Science, Constructor University, Campus Ring 1, 28759 Bremen, Germany*

E-mail: s.maity@ucl.ac.uk,smaity@constructor.university

S1 Equilibration of the System

The entire system contains more than $\sim$ 676K atoms and is solvated in a $\sim$ 19 nm $\times$ 19 nm $\times$ 17 nm simulation box. Initially, the system was energetically minimized and then a 2 ns NVT equilibration was performed at 300 K with the heavier atoms of protein, lipid, cofactors (heme, BChl, BPh, MQ8, UQ8, carotenoid), non-heme iron, and Ca^{2+} ions restrained. This was followed by a 5 ns NPT with the same restrictions. In the next 5 ns of NPT the Ca^{2+} ions were unrestrained, and in the following 10 ns the carotenoid molecules were released for the first 5 ns and the quinones MQ8 and UQ8 for the next 5 ns. In the next 5 ns-long NPT run the BChl and BPh pigments were not restrained any longer, followed by 5 ns with the lipid molecules unrestrained. The next 5 ns of NPT simulations had the protein of the LH1 ring restrained, and the following 5 ns had the β polypeptide of the LH1 complex restrained.

Finally, the last 10 ns of NPT was performed without any restraints. A 1-fs integration time step was used for these NVT and NPT simulations. The v-rescale thermostat was employed for the NVT equilibrium, while the Nose-Hoover thermostat[1] and the Parrinello-Rahman barostat[2] were used for NPT. Additionally, semi-isotropic pressure coupling was taken into account in NPT. The final structure after the classical equilibration was used as the starting structure for the QM/MM optimization, as described in the main text.

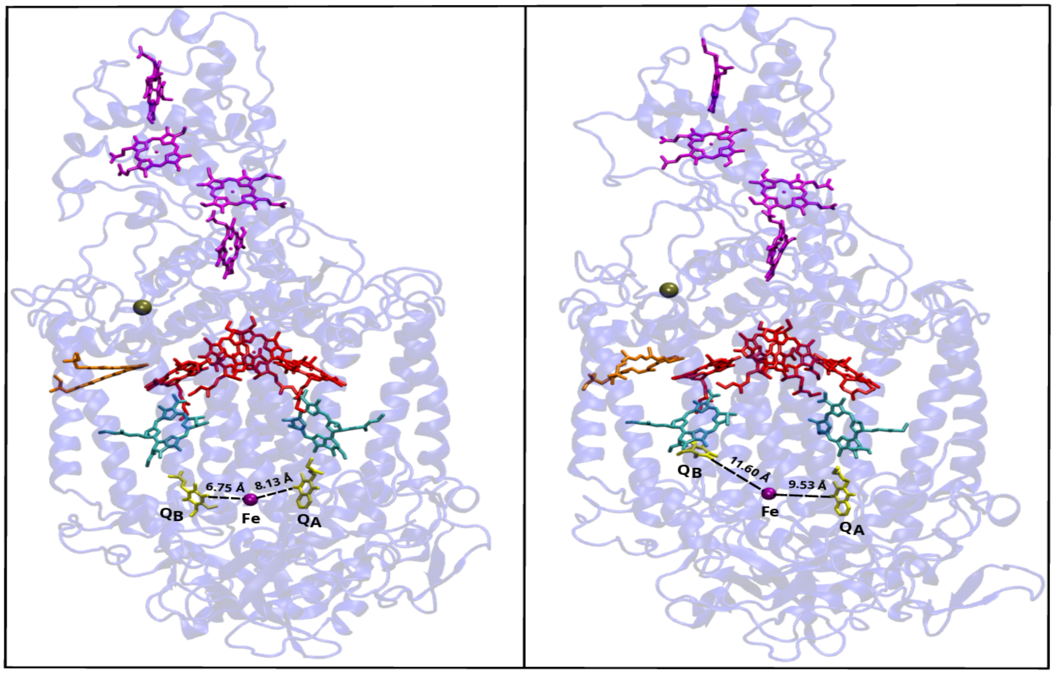

Figure S1: Structural shift of the quinone (Q_B) in the RC after the 200 ns simulations from the equilibration (right panel) compared to the crystal structure (left panel). The color scheme of the for pigment network is the same as shown in the main text.

We performed a 200 ns molecular dynamics (MD) simulation without restraints, saving frames every 10 ps to generate a total of 20,000 frames. These frames were used to calculate the excitation energies of all 32 BChl pigments in the LH1 complex, as well as 4 BChl and 2 BPh pigments in the RC, as described in the main text. Furthermore, structures were extracted every 50 ns from the same 200 ns trajectory and subjected to QM/MM minimization and TD-DFT excited state calculations in a QM/MM framework for charge transfer (CT) state analysis, as detailed in the main text. This analysis provides insights into the environmental effects on the CT state under thermal equilibrium conditions. Moreover,

after 200 ns of simulation, we observed a considerable displacement of the quinone Q_B from its position in the crystal structure, as illustrated in Fig. S1. This suggests that Q_B has a higher degree of dynamic mobility in its native protein environment, which may help it to transfer electrons and protons during the catalytic cycle. The movement may also reflect a shift between different binding states, a process that could be important in releasing reduced quinol and allowing the reaction center to continue operating within the respective photosystem[3]. However, this aspect falls outside the scope of the present study and will require further investigation.

S2 Summary of Computational Calculations

In this section, we provide a summary of the various types and starting structures of the calculations described in the main text and listed in Table S1. The table gives the initial structures studied along with the type of the associated ground state calculations, followed by the type of excited state calculations in QM/MM fashion. The respective results serve as a foundation for the further analysis of the charge transfer states and spectra.

Table S1: Overview of the different types and starting structures of the calculations performed in this study, including ground-state simulations (QM/MM optimization and QM/MM MD at the DFT (PBE-D3/DZVP-MOLOPT-GTH) level and classical MD simulations), excited-state calculations (TD-LC-DFT and TD-LC-DFTB), CT state analysis (for 2 BChls, 3 BChls and the L-Branch) and spectra.

**Ground State**	**Starting Structure**	**Excited State (QM/MM)**		**CT State Analysis**			**Spectra**
		**LC-DFT**	**LC-DFTB**	**2-BChl**	**3-BChl**	**L-Branch**	
QM/MM Opt.	After MD equilibration (1)	✓	✗	✓	✓	✓	✗
	At 50 ns from 200 ns MD (2)	✓	✗	✓	✗	✗	✗
	At 100 ns from 200 ns MD (3)	✓	✗	✓	✗	✗	✗
	At 150 ns from 200 ns MD (4)	✓	✗	✓	✗	✗	✗
	At 200 ns from 200 ns MD (5)	✓	✗	✓	✗	✗	✗
QM/MM MD	Result of QM/MM Opt. 1	✓	✗	✓	✗	✗	✗
	Result of QM/MM Opt. 2	✓	✗	✓	✗	✗	✗
	Result of QM/MM Opt. 3	✓	✗	✓	✗	✗	✓
	Result of QM/MM Opt. 4	✓	✗	✓	✗	✗	✗
	Result of QM/MM Opt. 5	✓	✗	✓	✗	✗	✗
Classical MD	200 ns after MD equilibration	✗	✓	✗	✗	✗	✓

S3 Comparison of Structures from 3WMM and 5Y5S

In this study, we used the structure with PDB code 3WMM, which has a resolution of 3.0 Å. By now, a second structure with PDB code 5Y5S[4] with a higher resolution of 1.9 Å is available. This structure slightly differs in the positions of bacteriochlorophylls, carotenoids, and the $\alpha\beta$ polypeptides in the LH1 complex, as well as in the calcium-binding site. In addition, 5Y5S includes multiple ubiquinones (QB), which help to reveal the quinone transport pathway through the LH1 complex.

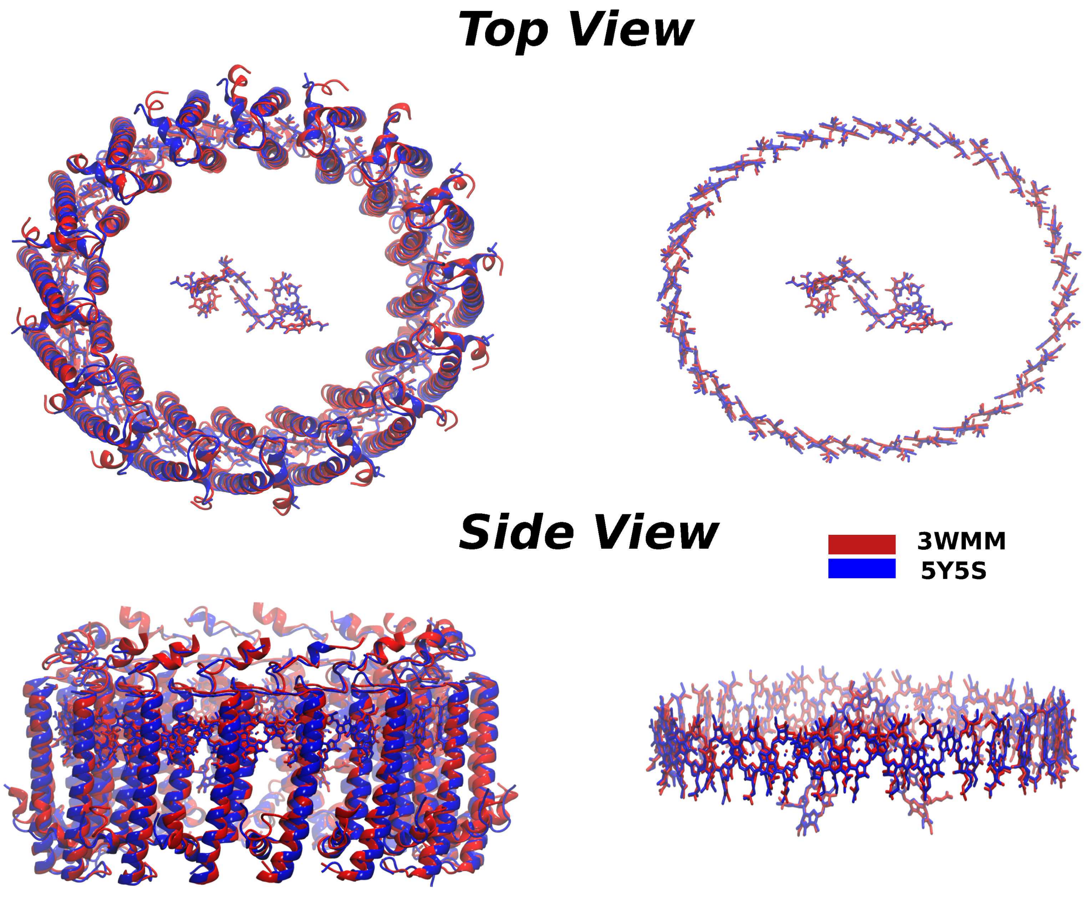

Figure S2: Structural alignment of the LH1-RC complex proteins and the associated BChl and BPh pigments between PDB entries 3WMM and 5Y5S.

To compare the two structures, we first aligned them using VMD[5]. Since both structures have the same number of residues resolved in the L-subunit of the RC, the alignment was based on this subunit. As shown in Fig. S2, the overall alignment is good. Although 5Y5S

includes some extra loop residues in the LH1 $\alpha\beta$ polypeptides, the rest of the structure still aligns well with 3WMM. The pigment molecules are also well-aligned between the two structures. Moreover, we first equilibrated the system using classical MD, then performed QM/MM optimization of the pigment pairs to run TD-DFT calculations for CT state analysis. Because of this procedure, the results for the present analysis between the two structures is likely very small. Only the loop regions of the structure 5Y5S might introduce some slightly modified long-range electrostatic effects.

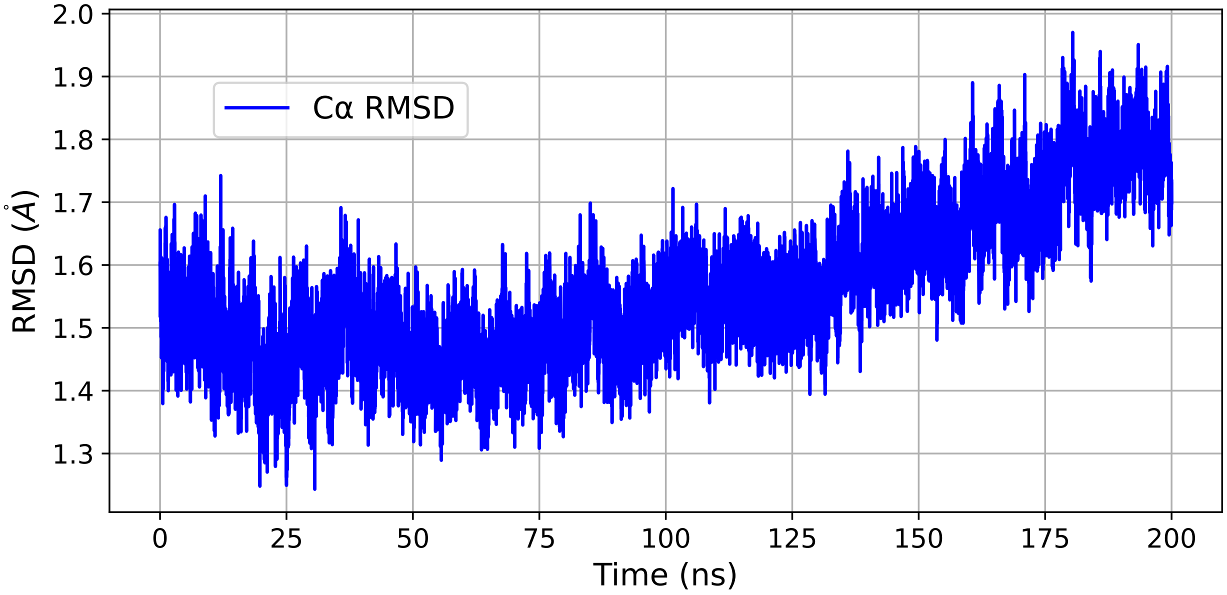

Figure S3: RMSD of the Cα atoms of the L-subunit over a 200 ns MD simulation of 3WMM, calculated with respect to the corresponding subunit in the crystal structure 5Y5S.

Since the calcium-binding sites in the structure 5Y5S add structural stability, we compared the RMSD of the Cα atoms in the L-subunit of the structure 3WMM along this trajectory with respect to the crystal structure 5Y5S, as shown in Fig. S3. The RMSD remains between 1–2 Å over the simulation period considered in this study, consistent with the value of 1.68 Å reported by Yu et al.[4] for these two crystal structures. These small deviations may induce minor changes in absorption properties due to the Ca binding sites, as suggested in Ref.[4], but are not expected to cause significant effects. Moreover, as discussed in the main text, the absorption peak anyway needs to be corrected for CT state effects and

TD-DFT shortcomings.

S4 Excitation Properties of the BChl Pigment Pair

In the main text, we presented a visual analysis of the lowest excitation state (S_1) and low-energy CT states for various BChl pairs including the special pair P_M/P_L and the adjacent pigments B_L and B_M across both the active and inactive branches, considering the presence and absence of the protein environment using QM/MM-optimized structures obtained after classical MD equilibration. This analysis was based on natural transition orbital representations and difference density plots (see Figs. 4 and 5 in the main text) after TD-DFT calculations as discussed in the main text. Here, we provided all the ten excited-state properties extracted for these pigment pairs in Table S2 (for P_L/B_L), Table S3 (for P_M/P_L), and Table S4 (for P_M/B_M), respectively.

Table S2: Excited-state properties of the P_L/B_L pair, including vertical excitation energies (VEE in eV), oscillator strengths (f) and natural transition orbital (NTO) contributions for the first 10 excited states, are presented for calculations with (QM/MM) and without (QM) the protein environment for the QM/MM optimized structure after classical equilibration. Only the two biggest NTO contributions are presented.

**With Protein (QM/MM)**				**Without Protein (QM)**		
**State**	**VEE**	**f**	**NTO**	**VEE**	**f**	**NTO**
S_1	1.656	0.00	1.0	1.999	0.19	0.60, 0.24
S_2	2.007	0.16	0.44, 0.40	2.022	0.89	0.59, 0.23
S_3	2.029	0.93	0.44, 0.39	2.312	0.13	0.59, 0.19
S_4	2.274	0.00	0.99	2.324	0.11	0.59, 0.19
S_5	2.290	0.15	0.62, 0.16	2.542	0.00	1.0
S_6	2.302	0.10	0.59, 0.18	2.572	0.00	1.0
S_7	2.952	0.00	0.99	3.164	0.00	0.97
S_8	3.164	0.00	0.95	3.172	0.00	0.98
S_9	3.213	0.00	0.94	3.240	0.00	1.0
S_{10}	3.231	0.00	0.97	3.242	0.00	0.99

Table S3: Same as Table S2 but for the special pair P_M/P_L.

With Protein (QM/MM)				Without Protein (QM)		
**State**	**VEE**	**f**	**NTO**	**VEE**	**f**	**NTO**
S_1	1.882	0.75	0.88	1.880	0.79	0.87
S_2	2.006	0.23	0.87	2.023	0.18	0.90, 0.10
S_3	2.030	0.19	0.67, 0.16	2.029	0.17	0.79, 0.10
S_4	2.160	0.10	0.93	2.154	0.09	0.97
S_5	2.266	0.08	0.62, 0.22	2.266	0.08	0.60, 0.20
S_6	2.290	0.13	0.45, 0.31	2.324	0.12	0.38, 0.36
S_7	2.537	0.00	0.97	2.644	0.00	0.97
S_8	2.732	0.00	0.98	2.779	0.00	0.98
S_9	2.925	0.00	0.98	3.119	0.00	0.93
S_{10}	3.062	0.00	0.98	3.135	0.00	0.80, 0.13

Table S4: Same as Table S2 but for the P_M/B_M pair.

With Protein (QM/MM)				Without Protein (QM)		
**State**	**VEE**	**f**	**NTO**	**VEE**	**f**	**NTO**
S_1	1.994	0.15	0.48, 0.36	1.994	0.15	0.48, 0.36
S_2	2.015	0.90	0.48, 0.35	2.015	0.0.90	0.48, 0.35
S_3	2.252	0.00	0.99	2.252	0.00	0.99
S_4	2.312	0.11	0.72, 0.23	2.312	0.11	0.72, 0.23
S_5	2.332	0.10	0.70, 0.25	2.332	0.10	0.70, 0.25
S_6	2.627	0.00	1.0	2.627	0.00	1.0
S_7	3.025	0.00	1.0	3.025	0.00	1.0
S_8	3.141	0.00	0.90	3.141	0.00	0.90
S_9	3.161	0.00	0.99	3.161	0.00	0.99
S_{10}	3.180	0.00	0.91	3.180	0.00	0.91

S5 Excitation Properties of Triple BChl Pigments

In addition to the two-pigment pairs, we also performed excitation profile analyzes for systems containing three BChl pigments. As discussed in the main text, the S_1 state and the low-lying CT states are illustrated using density difference analyses (see Fig. 9 in the main text) after TD-DFT calculations. Here, we report the ten lowest excited states for each

system based on QM/MM-optimized structures after classical MD equilibration. Table S5 presents the excitation properties of the $[P_M P_L]/B_L$ setup, while Table S6 provides the corresponding data for $[P_L P_M]/B_M$, both with and without the protein environment.

Table S5: Same as Table S2 but for the $[P_M P_L]/B_L$ three-pigment set.

With Protein (QM/MM)				Without Protein (QM)		
**State**	**VEE**	**f**	**NTO**	**VEE**	**f**	**NTO**
S_1	1.648	0.00	1.0	1.849	0.64	0.91
S_2	1.860	0.66	0.90	1.972	0.80	0.46, 0.40
S_3	1.931	0.01	0.99	1.997	0.14	0.73, 0.10
S_4	1.966	0.75	0.71, 0.17	2.038	0.17	0.33, 0.28
S_5	1.986	0.14	0.55, 0.31	2.192	0.06	0.60, 0.21
S_6	2.039	0.23	0.34, 0.25	2.255	0.06	0.59, 0.21
S_7	2.176	0.07	0.88, 0.10	2.314	0.15	0.68, 0.22
S_8	2.216	0.06	0.61, 0.25	2.318	0.08	0.39, 0.32
S_9	2.246	0.00	1.0	2.504	0.00	1.0
S_{10}	2.284	0.10	0.47, 0.30	2.532	0.00	1.0

Table S6: Same as Table S5 but for the $[P_L P_M]/B_M$ three-pigment set.

With Protein (QM/MM)				Without Protein (QM)		
**State**	**VEE**	**f**	**NTO**	**VEE**	**f**	**NTO**
S_1	1.872	0.91	0.86	1.879	0.97	0.84
S_2	1.965	0.54	0.60, 0.15	1.978	0.46	0.52, 0.20
S_3	2.011	0.26	0.84, 0.10	2.035	0.20	0.80, 0.10
S_4	2.044	0.05	0.55, 0.20	2.048	0.05	0.62, 0.22
S_5	2.148	0.10	0.94	2.132	0.11	0.70, 0.93
S_6	2.214	0.07	0.64, 0.19	2.254	0.08	0.61, 0.18
S_7	2.276	0.12	0.75, 0.22	2.318	0.10	0.44, 0.32
S_8	2.286	0.12	0.40, 0.36	2.330	0.07	0.50, 0.46
S_9	2.340	0.00	1.0	2.333	0.04	0.71, 0.26
S_{10}	2.467	0.00	1.0	2.550	0.00	1.0

S6 Excitation Properties of the Active L-Branch

Furthermore, we extended our analysis to include a numerically demanding four-pigment system comprising P_M, P_L, B_L, and H_L in the active L-branch, following a similar approach as used for the two- and three-pigment systems. As before, TD-DFT calculations were carried out on the QM/MM-optimized structure obtained after classical MD equilibration. The corresponding difference density plots, illustrating the results with and without the protein environment, are shown in Fig. 10 of the main text. The excited-state properties for the ten lowest extracted states under both conditions are summarized in Table S7.

Table S7: Same as Table S2 but for Active L-branch including P_M, P_L, B_L, H_L pigments.

With Protein (QM/MM)				Without Protein (QM)		
**State**	**VEE**	**f**	**NTO**	**VEE**	**f**	**NTO**
S_1	1.761	0.00	1.0	1.871	0.84	0.88
S_2	1.880	0.89	0.87	1.964	0.86	0.55, 0.16
S_3	1.969	0.79	0.50, 0.30	2.014	0.23	0.83, 0.10
S_4	2.004	0.13	0.72, 0.12	2.034	0.07	0.40, 0.26
S_5	2.041	0.15	0.36, 0.28	2.053	0.29	0.43, 0.23
S_6	2.054	0.00	0.98	2.203	0.06	0.97
S_7	2.080	0.36	0.70, 0.15	2.257	0.06	0.60, 0.20
S_8	2.153	0.00	1.0	2.286	0.02	0.94
S_9	2.191	0.09	0.78, 0.19	2.319	0.13	0.39, 0.36
S_{10}	2.221	0.03	0.58, 0.32	2.326	0.07	0.77, 0.20

References

(1) Evans, D. J.; Holian, B. L. The Nose–Hoover Thermostat. *J. Chem. Phys.* **1985**, *83*, 4069–4074, DOI: 10.1063/1.449071.

(2) Parrinello, M.; Rahman, A. Polymorphic Transitions in Single Crystals: A New Molecular Dynamics Method. *J. Appl. Phys.* **1981**, *52*, 7182–7190, DOI: 10.1063/1.328693.

(3) Kimura, Y.; Tani, K.; Madigan, M. T.; Wang-Otomo, Z.-Y. Advances in the Spectroscopic and Structural Characterization of Core Light-Harvesting Complexes from Purple Phototrophic Bacteria. *J. Phys. Chem. B* **2023**, *127*, 6–17, DOI: 10.1021/acs.jpcb.2c06638.

(4) Yu, L.-J.; Suga, M.; Wang-Otomo, Z.-Y.; Shen, J.-R. Structure of Photosynthetic LH1-RC Supercomplex at 1.9 Angstrom Resolution. *Nature* **2018**, *556*, 209–213, DOI: 10.1038/s41586-018-0002-9.

(5) Humphrey, W. F.; Dalke, A.; Schulten, K. VMD – Visual Molecular Dynamics. *J. Mol. Graph.* **1996**, *14*, 33–38, DOI: 10.1016/0263-7855(96)00018-5.
